# HIV-1 envelope glycoprotein stimulates viral transcription and increases the infectivity of the progeny virus through the manipulation of cellular machinery

**DOI:** 10.1038/s41598-017-10272-7

**Published:** 2017-08-25

**Authors:** Xiaozhuo Ran, Zhujun Ao, Adriana Trajtman, Wayne Xu, Gary Kobinger, Yoav Keynan, Xiaojian Yao

**Affiliations:** 1 Laboratory of Molecular Human Retrovirology, University of Manitoba, Winnipeg, MB Canada; 2 Department of Medical Microbiology, Max Rady College of Medicine, Rady Faculty of Health Sciences, University of Manitoba, Winnipeg, MB Canada; 3 Department of Microbiology, School of Basic Medical Sciences, Central South University, Changsha, Hunan 410078 P.R. China; 4 Research Institute in Oncology and Hematology, CancerCare Manitoba, Winnipeg, MB Canada; 5 Centre de Recherche en Infectiologie de l’Université Laval/Centre Hospitalier de l’Université Laval (CHUL), Québec, QC Canada

## Abstract

During HIV infection, large amounts of progeny viral particles, including infectious virus and a large proportion of defective viral particles, are produced. Despite of the critical role of the infectious viruses in infection and pathogenesis *in vivo*, whether and how those defective viral particles, especially the virus-associated envelope glycoprotein (vEnv), would impact viral infection remains elusive. In this study, we investigated the effect of vEnv on HIV-infected T cells and demonstrated that the vEnv was able to stimulate HIV transcription in HIV-infected cells, including peripheral blood mononuclear cells (PBMCs) isolated from HIV patients. This vEnv-mediated HIV transcription activation is mediated primarily through the interaction between vEnv and CD4/coreceptors (CCR5 or CXCR4). Through transcriptome analysis, we found that numerous cellular gene products involved in various signaling pathways were modulated by vEnv. Among them, we have further identified a cellular microRNA miR181A2, which is downregulated upon vEnv treatment, resulting in increased HIV LTR histone H3 acetylation and HIV transcription. Furthermore, we also found a vEnv-modulated cellular histone deacetylase, HDAC10, whose downregulation is associated with the increased infectivity of progeny viruses. Altogether, these findings provide evidence of the important role vEnv plays in modulating cellular environments and facilitating HIV expression and infection.

## Introduction

It is well known that during HIV infection, infected cells produce not only infectious viruses but also large amounts of noninfectious or defective particles as a result of highly reading-frame-error-prone reverse transcriptase^1, 2^. Some studies estimate that the ratio of infectious to noninfectious particles is between 1 in 1,000 and 1 in 60,000 depending on the measurement methods^2–4^. While the small proportion of infectious viruses continues their infection and dissemination, the physiological role of the large amounts of defective particles present during infection is still not fully explored. Some studies have shown that these defective particles are able to stimulate CD4 + T lymphocytes and induce their apoptosis, which contribute to HIV infection and pathogenesis^5–9^.

During natural infection, HIV envelope glycoproteins (gp120 and gp41) are present as trimers on the surface of viral particles^10–12^, and their binding to cell receptor CD4 and coreceptors (CCR5 or CXCR4) is essential for virus entry into CD4 + T lymphocytes, monocytes and macrophages^13–15^. Meanwhile, the interaction of gp120 with CXCR4 has been shown to be able to activate the cellular actin-depolymerizing factor cofilin and lead to actin skeleton rearrangement, which may facilitate the process of viral integration^16^. In addition to directly acting on early HIV infection, some *in vitro* studies have also documented that the interaction of a recombinant gp120 and CD4/co-receptors led to activation of cellular pathways including cell adhesion, proliferation and actin modulation^7, 17, 18^, and the elevated expression of cytokines and chemokines^18^; Other studies also suggest that gp120 could interfere with CD4 costimulatory functions and induces apoptosis^19^. All of these studies provide evidence of the important role of HIV gp120 in the establishment of HIV infection and its induced pathogenesis. However, there are still several key questions remaining: What is the effect of vEnv on viral expression in HIV-infected CD4 + T cells, especially for those latently infected T cells? Can vEnv modulate the infectivity of the newly produced progeny viruses from those HIV infected T cells?

In this study, we sought to investigate the effects of vEnv on HIV transcription, and its impact on the infectivity of newly produced progeny viruses. To elucidate the molecular mechanisms underlying these vEnv’s activities, we also performed transcriptome sequencing and more detailed biochemical analysis and the results revealed that some cellular signaling pathways and/or cofactors are participating in these biological activities of vEnv. Furthermore, we have identified that a cellular microRNA 181A2 (miR181A2), which is downregulated upon vEnv treatment and its downregulation resulted in an upregulated PCAF (cellular p300/CBP associated factor) expression, and enhanced LTR-associated histone H3 acetylation and HIV transcription. Also we have demonstrated HIV vEnv was able to suppress the cellular histone deacetylase 10 (HDAC10) expression and its downregulation led to an increased infectivity of the produced progeny virus.

## Results

### HIV non-infectious virus-associated envelope glycoprotein (vEnv) stimulates HIV LTR-driven gene expression

**Figure 1:**
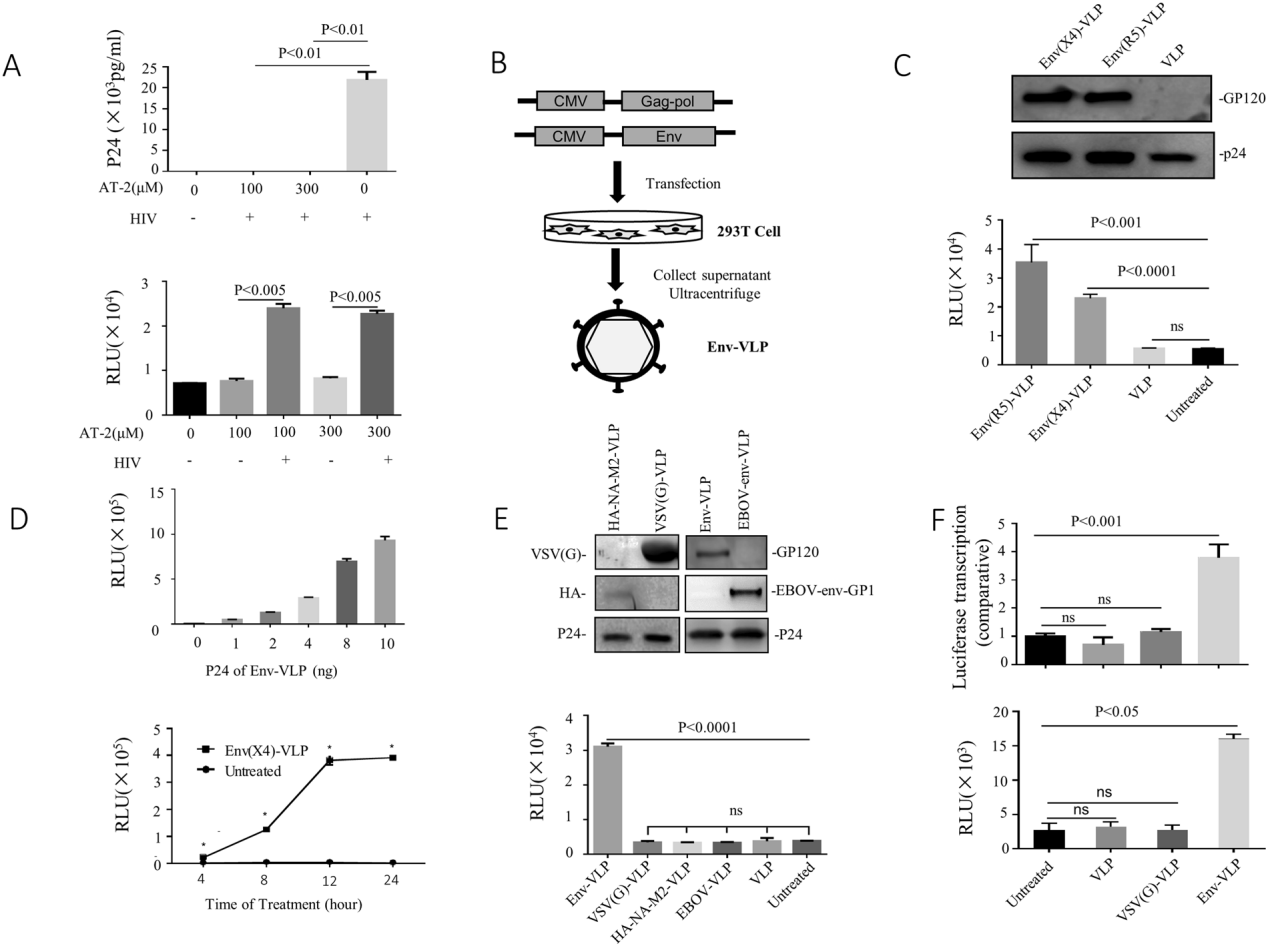
HIV-1 virus-associated Env (vEnv) activates LTR-driven gene expression. (**A**) Upper panel: Detection of infectivity of AT-2-treated HIV. Wild-type HIV virus was treated with the indicated concentrations of AT-2 for 1 hour at 37 °C and used to infect C8166 cells for three days. The p24 level in the supernatant was detected by p24 ELISA (n = 2). Lower panel: Luciferase expression in TZMb1 cells infected with AT-2-treated virus for 24 hours (n = 2). (**B**) Schematic for producing HIV Env-VLP. HIV Gag-pol (Δ8.2) and Env (X4/R5-tropic) plasmids were co-transfected into 293 T cells; after 48 hours, Env-VLP in the supernatant was collected and concentrated by ultracentrifugation. Purified Env-VLPs were used to treat various cells. (**C**) Upper panel: Western blot confirming the presence of gp120 and p24 of Env-VLP. Lower panel: Luciferase expression in TZMb1 cells treated with Env(X4)-VLP, Env(R5)-VLP or VLP or untreated for 24 hours (n = 3). (**D**) Upper panel: Luciferase expression in TZMb1 cells treated with varying amounts (0-10 ng) of Env(X4)-VLP (n = 3). Lower panel: Luciferase expression in TZMb1 cells treated with Env(X4)-VLP for different periods (n = 3). (**E**) Effect of various viral glycoproteins on HIV transcription. Upper panel: Detection of the presence of different viral glycoprotein in HIV VLP by western blotting. Each VLP stock was lysed, and glycoproteins were detected using corresponding antibodies (data in the right panel and the left panel are from two experiments). Lower panel: Luciferase expression was detected in TZMb1 cells treated with Env-VLP, VSVG-VLP, HA-NA-M2, EBOLA-VLP or VLP (without Env) or untreated for 24 hours, and luciferase activity was measured (n = 3). (**F**) Luciferase comparative transcription (luciferase/GAPDH, n = 3) in TZMb1 cells treated with Env-VLP, VSVG-VLP, VLP (without Env) or untreated (upper panel). Luciferase activity was measured in TZMb1 cells treated with Env-VLP, VSVG-VLP or VLP or untreated cells (n = 2) (Lower panel). Data are the mean and sd. Ns, not significant p > 0.05; *p < 0.05. (two-tailed unpaired t-test; multiple-t test; correction for multiple comparison using the Holm-Sidak method)

During HIV infection, the majority of produced progeny viruses are noninfectious and are termed “defective particles.” Although these defective viruses are not able to infect hosts, they are far from innocuous^1^. To investigate whether these noninfectious viral particles play any role in HIV-infected cells, we first treated HIV viruses (N119) with aldrithiol-2 (AT-2), which can inactivate viruses by preferential covalent modification of internal viral proteins (NC) while preserving the structural and functional properties of the viral envelope protein^20^, and used them to infect C8166 T cells. The results showed that AT-2-treated virus lost its infectivity (Fig. 1A upper panel). Meanwhile, these AT-2 viral particles were used to treat TZMb1 cells, which express CD4, CCR5 and CXCR4 and contain a reporter gene firefly luciferase (Luc) driven by HIV LTR^21^. Interestingly, these inactivated viruses were shown to be able to activate HIV LTR-controlled Luc expression (Fig. 1A lower panel), suggesting a stimulating effect on HIV-LTR driven transcription in TZMb1 cells.

To identify the main determinant in viral particles for the activation of HIV-1 LTR, we first produced envelope glycoprotein-incorporated HIV virus-like particles (Env-VLP) by co-transfecting 293 T cells with HIV × 4- or R5-tropic envelope glycoprotein-expressing plasmid and HIV-packaging plasmid (Del-8.2), as described previously^22^ (Fig. 1B). The presence of × 4- or R5-tropic vEnv on purified VLPs was confirmed by western blot analysis using anti-gp120 and anti-p24 antibodies, respectively (Fig. 1C upper panel). To test whether × 4- or R5-tropic Env-VLP are able to stimulate HIV LTR-derived transcription, TZMb1 cells were treated with equal amounts of × 4- or R5- Env-VLP, and the HIV LTR-driven Luc activity was measured after 24 hours. The results showed that both × 4- or R5-Env-VLP induced 7-fold- or 4-fold-increased Luc activity, respectively. In contrast, VLP lacking Env had no effect on HIV LTR-driven Luc expression (Fig. 1C, lower panel). We also showed that the Env-VLP-mediated stimulating effect was dose- and time-dependent (Fig. 1D), indicating that both × 4- and R5-envelope glycoproteins may be the main determinants in HIV VLP responsible for HIV LTR activation,

We next tested whether other viral envelope proteins could also exhibit similar effects on HIV LTR-derived gene expression. The virus entry-competent VSV-G, H5N1 or Ebola (Mayinga strain) envelope glycoprotein-pseudotyped HIV VLPs were also produced as described previously^23, 24^, and the VLP-incorporated glycoproteins were detected by western blotting (Fig. 1E, upper panel). Equal amounts of each Env pseudotyped VLP were used to treat the TZMb1 cells. Interestingly, we observed that only HIV Env-VLP induced significantly high Luc activity (Fig. 1E, lower panel), while no other viral Env-VLP demonstrated any stimulating effect on HIV LTR, indicating a unique and specific effect of HIV-1 Env glycoprotein on LTR-driven expression.

Furthermore, we tested whether Env-VLP acts on HIV LTR transcription. To do so, TZMb1 cells were treated with HIV Env-VLP, and after 24 hours of treatment, Luc gene mRNA was measured using RT-PCR. Treatment with HIV Env-VLP, but not VSV Env-VLP, induced a 4-fold increase in Luc mRNA (Fig. 1F, upper panel) in TZMb1 cells, which is correlated with elevated levels of Luc activity (Fig. 1F, lower panel). Altogether, these data indicate that HIV envelope glycoprotein is the main determinant that stimulates HIV LTR-driven gene transcription and expression.

### The interaction between vEnv and CD4/coreceptors (CCR5 and CXCR4) is essential for HIV transcription activation

**Figure 2:**
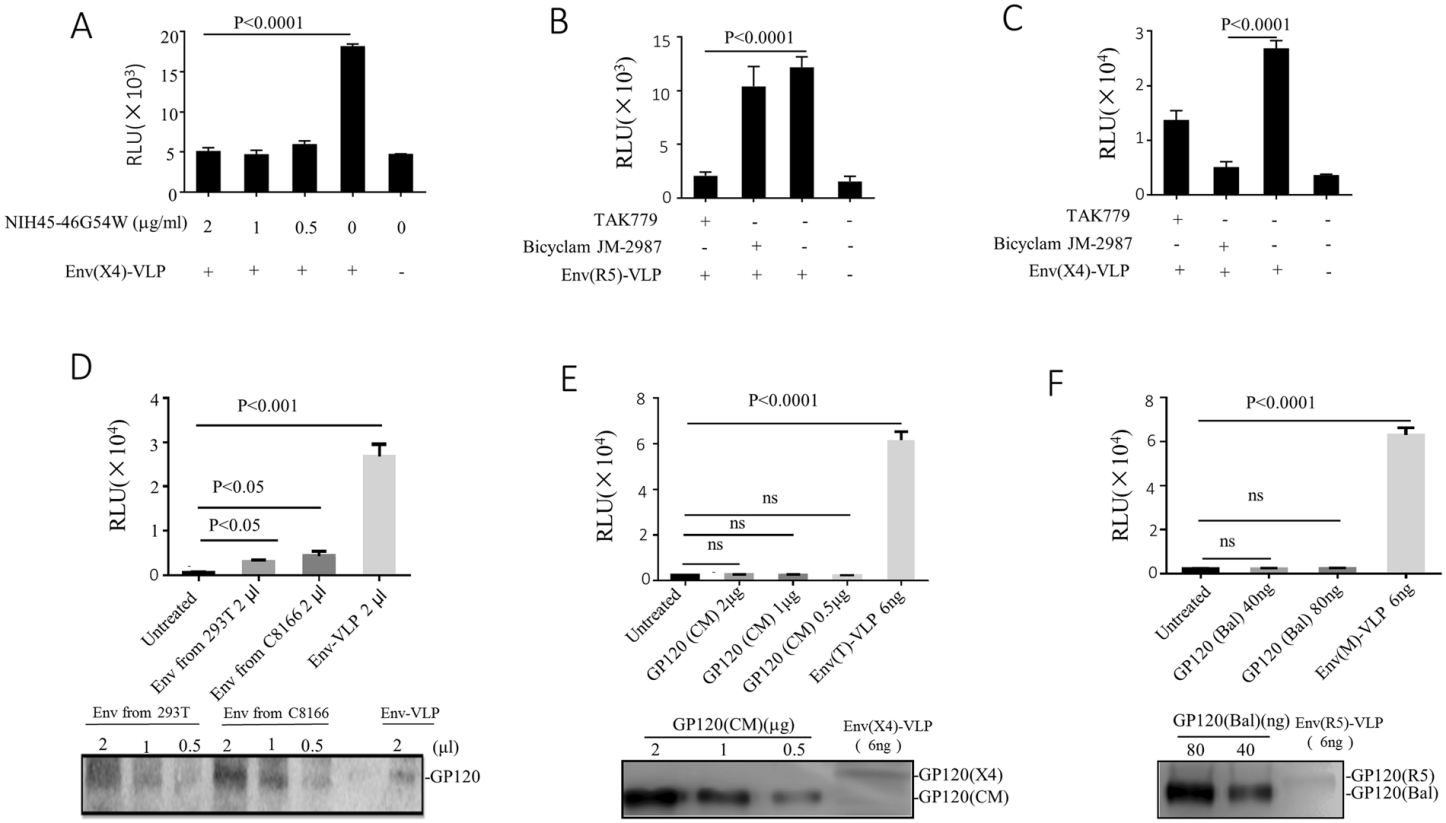
Interaction between HIV virus-associated envelope glycoprotein (vEnv) and CD4/coreceptors is essential for HIV transcription activation. (**A**) Expression of luciferase in TZMb1 cells treated with Env-VLP in presence of different concentrations of an anti-gp120 neutralizing antibody, which targets the interaction region between CD4 and gp120. After 24 hrs, luciferase activity in the TZMb1 cells was detected (n = 3). TZMb1 cells were first cultured in the presence of CCR5 inhibitor TAK779 or CXCR4 inhibitor Bicyclam JM-2987 for 2 hr. Then cells were treated with Env(R5)-VLP (**B**) or Env(X4)-VLP (**C**) in the presence of CCR5 inhibitor TAK779 or CXCR4 inhibitor Bicyclam JM-2987. After 24 hours, cells were collected, lysed and used to measure luciferase activity (n = 4). TZMb1 cells treated with Env(X4 or R5)-VLP, shed Env proteins (**D**), recombinant gp120 (CM) (**E**), or the recombinant gp120 (Bal) (**F**). After 24 hours, cells were collected, lysed and used to measure luciferase activity (n = 3). Data are the mean and sd. Ns, not significant p > 0.05 (two-tailed unpaired t-test).

It is well accepted that in addition to mediating membrane fusion, the interaction of HIV-1 gp120 with its chemokine coreceptor (CCR5 /CXCR4) also triggers cellular signal transduction^25^. We sought to determine whether the interaction of vEnv with CD4/coreceptors is also required for the activation of HIV transcription. Briefly, Env(X4)-VLPs were incubated with TZM-b1 cells in the presence of a highly potent neutralizing HIV-1 antibody NIH45-46^G54W^, which specifically targets the CD4 binding site of the HIV-1 envelope^26^. Luc activity was measured after 24 hours. NIH45-46^G54W^, in concentrations ranging from 0.5–2 μg/ml, completely inhibited Env(X4)-VLP-induced Luc activities (Fig. 2A), indicating that the gp120/CD4 interaction is essential for the action of vEnv on HIV LTR activation. Meanwhile, we treated TZM-b1 cells with a CCR5 chemokine receptor antagonist TAK-779^27^ or a CXCR4 inhibitor Bicyclam JM-2987^28^ for 2 hrs prior to incubating the cells with × 4- or R5-tropic Env-VLP. The results revealed that TAK-779 almost completely inhibited the activating effect of Env(R5)-VLP (Fig. 2B) but had a moderate effect on the action of Env(X4)-VLP (Fig. 2C). In contrast, Bicyclam JM-2987 had no effect on Env(R5)-VLP (Fig. 2B) but significantly decreased the Luc activity induced by Env(X4)-VLP (Fig. 2C). The mechanism by which TAK-779 could partially block Env(X4)-VLP-mediated activation is still unclear. Nevertheless, our above results clearly indicate that the interactions of gp120 with CD4/coreceptors (CCR5 and CXCR4) are necessary for vEnv-induced HIV transcription.

### Virus-associated Env glycoprotein is a significantly more effective activator of HIV transcription than shed or recombinant HIV gp120 proteins

It is known that soluble gp120 (sgp120) is shed and present in patients’ serum *in vivo* ^29^. Given the ability of HIV-1 vEnv to activate HIV transcription, we next asked whether HIV sgp120 or some recombinant envelope proteins could also act on HIV transcription. First, we compared vEnv and sgp120 for their effects on viral transcription. Briefly, the sgp120 from transfected 293 T cells or HIV (pNL4.3)-infected C8166T cells were obtained as described in Materials and Methods and detected by western blotting (Fig. 2D, lower panel). Next, sgp120 was added to TZMb1 cells for 24 hours. In parallel, an equal volume of Env(X4)-VLP was used as a control. The results showed that while Env(X4)-VLP induced a significant increased Luc activity (p < 0.001), the Luc activity produced from the cells treated with sgp120 was only moderately increased (p < 0.05), (Fig. 2D, upper panel). These results suggest that sgp120 may only have a limited effect on HIV transcription.

We also tested the effect of recombinant HIV-1 gp120 (CM) or HIV-1 gp120 (Bal) on HIV transcription, as these recombinant gp120s have been reported to be capable of inducing apoptosis^30, 31^. Briefly, TZMb1 cells were treated with different concentrations of recombinant gp120 (CM) (Fig. 2E) or recombinant gp120 (Bal) (Fig. 2F) for 24 hours, and the Env(X4)-VLP or Env(R5)-VLP were used as positive controls. The results showed that neither r-gp120 could activate HIV LTR gene expression (Fig. 2E,F, upper panels), even at much higher input amounts of r-gp120 compared to vEnv (Fig. 2E,F, lower panels). Notably, the molecular weight of both r-gp120 proteins was less than 120 kDa, probably due to varied glycosylation in different production systems. Nevertheless, these data indicate that vEnv, but not the sgp120 or recombinant gp120 tested in this study, has a significant effect on HIV LTR transcription in TZMb1 cells.

### HIV vEnv induces viral transcription in HIV-latently-infected CD4 + T cells (J-Lat6.3), HIV-infected non-stimulated PBMCs and PBMCs from ART-treated patients

**Figure 3:**
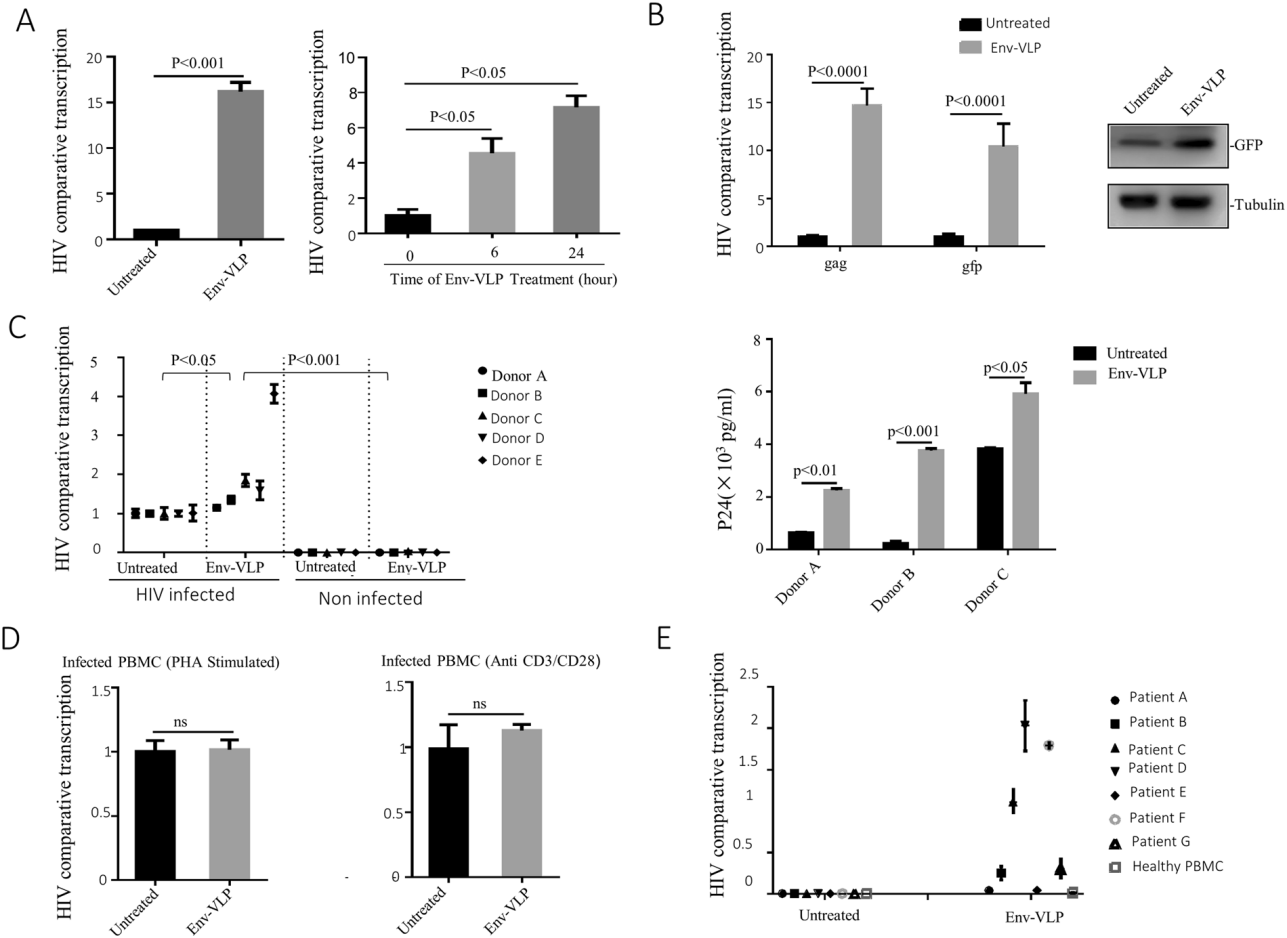
HIV vEnv induced viral transcription in HIV-infected CD4 T cell line (J-Lat 6.3), HIV-infected non-stimulated PBMCs, and latent-infected PBMCs in ART-treated patients. (**A**) Increased transcription of HIV gag in J-Lat 6.3 T cells treated with Env(X4)-VLP. J-Lat 6.3 T cells were treated or non-treated with Env(X4)-VLP for 24 hrs. HIV gag mRNA was detected by RT-PCR, normalized against the housekeeping gene GAPDH (n = 3), and expressed as comparative transcription level. (**B**) J-Lat 6.3 T cells were treated with Env(X4)-VLP for 0, 6, and 24 hrs (as indicated) and after 24 hrs, cells were lysed and HIV comparative transcription levels (gag/GAPDH) and the reporter gene GFP comparative transcription levels (GFP/GAPDH) were detected by RT-PCR (left panel). Also, the Env(X4)-VLP treated or untreated J-Lat 6.3 T cells were lysed and the expression of GFP was detected by western blot with corresponding antibody (right panel). (**C**) HIV comparative transcription levels in HIV-infected resting PBMCs treated with Env(X4)-VLP or untreated (n = 3). PBMCs were isolated from five donors and infected with HIV virus for 24 hrs without stimulation and the infected PBMCs were washed and kept in culture medium for 2 days. Then, cells were treated with Env(X4)-VLP or untreated. Meanwhile, the untreated HIV-infected PBMCs, untreated and uninfected PBMCs or Env(X4)-VLP-treated uninfected PBMCs were used as controls. After 24 hours of treatment, HIV comparative transcription levels (gag/GAPDH) were detected by RT-PCR (left panel). Meanwhile, the infected resting PBMCs treated or untreated with Env-VLP, (as described in left panel) were co-cultured with C8166 T cells for three days. Then, the HIVp24 levels in the supernatant of co-cultures were measured by anti-HIVp24 ELISA (right panel). (**D**) HIV comparative transcription levels were detected in PHA or anti-CD3/CD28 stimulated HIV-infected PBMCs followed by the treatment with Env-VLP or not (n = 3). (**E**) HIV comparative transcription levels in non-stimulated PBMCs isolated from seven HIV latent patients, that were treated or non-treated with Env(X4)-VLP for 24 hrs, and then the HIV gag mRNA was detected by RT-PCR (n = 3). Data are the mean and sd. Ns, not significant p > 0.05. (two-tailed unpaired t-test)

To specifically test whether vEnv induces HIV transcription in HIV-infected cells, we first tested the action of vEnv in J-Lat 6.3 T cells that harbor an Env-defective HIV provirus containing the green fluorescent protein (GFP) in the *nef* region, which has been widely used as a HIV latently infected cell model^32^. Upon treatment of HIV Env(T)-VLP, HIV transcription was monitored by viral mRNA or GFP expression. J-Lat 6.3 T cells were treated with Env(X4)-VLP for 24 hours, the total mRNA was isolated and the gag mRNA and GFP mRNA levels were measured using RT-PCR. The gag mRNA expression increased 14- to 16-fold in Env(X4)-VLP-treated cells compared to mock-treated cells (Fig. 3A, left panel). As expected, six hours of treatment with a lower dose of Env(X4)-VLP resulted in a significantly increased mRNA level (Fig. 3A, right panel). A higher mRNA and protein level in HIV LTR-driven GFP was detected in Env(X4)-VLP-treated cells by RT-PCR and Western blotting (Fig. 3B). These data suggest that vEnv is able to stimulate HIV transcription in HIV latently infected J-Lat 6.3 T cells.

Next, we explored whether vEnv could induce HIV transcription in HIV-infected primary PBMCs. To address this question, we mimicked HIV infection in two different ways. First, we isolated PBMCs from five healthy donors and infected them with HIV-1 (N119 strain) overnight^33, 34^. Three days later, infected PBMCs were treated with Env(X4)-VLP, and 24 hours later, cells were collected and HIV mRNA in the cells was measured using RT-PCR. The results showed that compared with the infected and mock-treated cells, HIV transcription was increased at various levels in the infected PBMCs treated with Env(X4)-VLP (Fig. 3C, left panel). We also detected significantly greater infectious virus production from the vEnv-treated HIV infected resting PBMCs by co-culturing with C8166 T cells (Fig. 3C right panel). These results indicate that vEnv is able to induce viral transcription in infected primary resting PBMCs. Meanwhile, we tested whether vEnv could affect on HIV transcription in PHA- or anti-CD3/CD28-stimulated HIV-infected PBMCs. The results showed no significant difference in vEnv-treated and untreated groups (Fig. 3D), suggesting that vEnv could not further stimulate viral transcription in PHA or anti-CD3/CD28 pre-stimulated HIV-infected PBMCs.

The above observations indicate that vEnv is able to induce HIV transcription in the HIV-infected J-Lat 6.3 T cell line and unstimulated human PBMCs. We then asked whether vEnv activates HIV transcription in resting PBMCs isolated from combination antiretroviral therapy (cART)-treated aviremic HIV-infected patients. Seven patients were selected based on the following criteria: HIV-1 infected; currently treated with cART; a plasma HIV-1 RNA <50 copies per ml for at least six months; and a CD4 count >300 per ml. Following isolation of PBMCs without stimulation, cells from each donor were treated with Env(X4)-VLP or mock-treated. After 48 hours of treatment, cells were collected and HIV transcription was analyzed by detecting the gag mRNA level via RT-PCR. Interestingly, the results revealed the vEnv treatment led to an increased HIV transcription in PBMCs from 5 of 7 patients, with a maximum 2-fold higher transcription than in the mock-treated group (Fig. 3E).

### vEnv induced HIV transcription activation is associated with alteration of multiple cellular pathways

Because HIV vEnv is able to induce viral transcription in both HIV**-**infected non-stimulated PBMCs and latently infected PBMCs from cART-treated patients, it becomes interesting to explore its underlying mechanism. Therefore, we utilized whole-RNA transcriptome sequencing to analyze the overall gene expression in J-Lat 6.3 T cells upon treatment of vEnv. Briefly, J-Lat 6.3 T cells were treated with Env(X4)-VLP or untreated for 24 hours and the total RNA was isolated, reverse transcribed into cDNA and processed using total RNA profile analysis.

**Figure 4:**
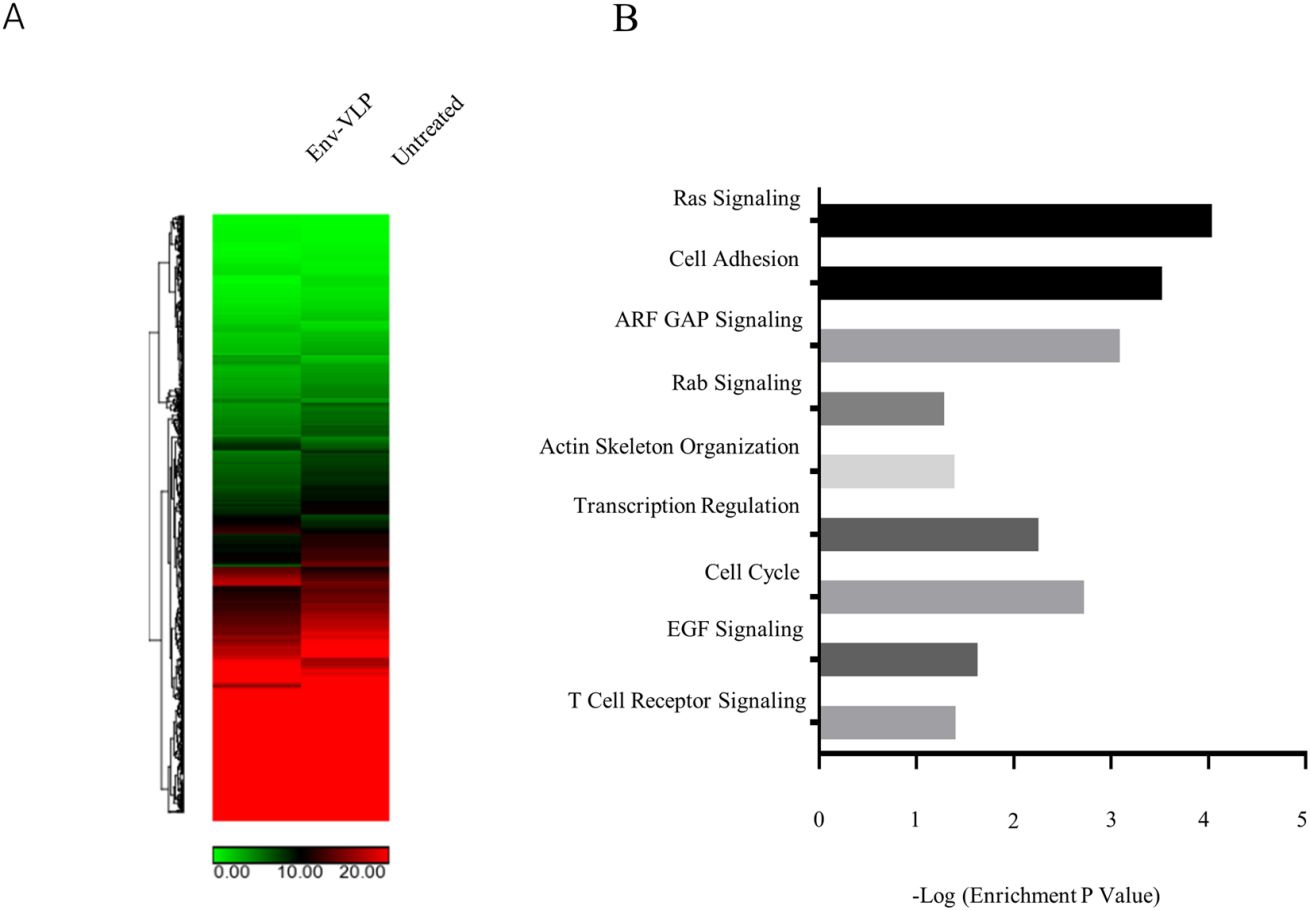
Treatment of vEnv(X4) of J-Lat 6.3 T cells induces the transcriptional changes of multiple cellular genes involved in different signaling pathways. (**A**) Heatmap of genes (p < 0.05) in J-Lat 6.3 T cells untreated (n = 2) or treated with Env(X4)-VLP (n = 3) in technical replicates showing hierarchical clustering. (Lifescope v2.5.1 software from Life Technologies with the 2-mismatch setting). (**B**) Enrichment of genes modulated by vEnv. The fold enrichment was calculated based on the frequency of genes annotated to the term compared to their frequency in the genome in the DAVID Bioinformatics database.

The results of RNA-Seq analysis showed that among 28,802 transcripts, there were 1,349 differentially expressed genes whose expression levels had significant changes (p < 0.05) in the Env(X4)-VLP treated group. In the heat map, the hierarchical clustering of RNA-seq data from J-Lat 6.3 T cells treated with Env(X4)-VLP or mock-treated demonstrated their distinct genome-wide expression profiles (Fig. 4A).

We investigated the 1,349 genes’ functions using the KEGG and DAVID bioinformatics resources^34, 35^ and divided them into groups according to their known functions and involved pathways (Fig. 4B). This gene analysis revealed that vEnv treatment modulates the expression of genes involved in multiple cellular signaling pathways, including transcription regulation, actin skeleton organization, and T cell receptor signaling, as well as various microRNAs and many genes encoding proteins that have been reported to be directly or indirectly regulated by HIV viral proteins (Table 1). Some upregulated gene encoding proteins were shown to be regulated by HIV glycoprotein gp120 (CD2, CD40L, CCR5, FN1, MANEA, SDC2, TJP1 and UBE2D1), gp41 (AP1S2, AP3M1, FN1), Tat (TAF9, LAMC1, MED21, SDC2, CCR5, CDK8, FN1), Vpr (NUP54), Protease (PLS1) and Nef (CD40L, AP1S2, AP3M1, CCR5, FN1). These genes are usually associated with cell surface receptors, adhesion molecules and RNA polymerase II positive regulators. Among the downregulated genes, several genes encoded proteins that were previously shown to be regulated by viral protein gp120 (FCAR, ESR2, GAA, NFKBIB, TNFRSF10D), gp41 (ARHGEF1, GAA), Vpr (MKNK2), and Tat (EEF1D, NFKBIB). These cellular genes are usually negative regulators or host immune receptors (as summarized in Table 1).

**Table 1:** Genes encoding proteins associated with HIV proteins +, upregulated; −, downregulated.

Gene Name	Regulation	Functional Category	Proposed Role in HIV Replication
CD2	+	Cell adhesion molecules	HIV-1 latency
CD40L	+	TNF superfamily member	Immunological control of viral replication
TAF9,CDK8,MED21	+	Facilitate RNAP II complex function	Regulate Tat mediated HIV-1 transactivation;
AP1S2	+	mediates the recruitment of clathrin	Interact with HIV-1 gp41 or Nef protein
AP3M1	+	Facilitates the budding of vesicles	Viral Budding
CCR5	+	Chemokine receptor	Viral entry, Chemokine signaling pathway
FN1	+	Bind to integrin	Binds to HIV-1 gp120/160 and increase infection
LAMC1	+	extracellular matrix glycoproteins	Upregulated by HIV infection or Tat protein
MANEA	+	Endomannosidase	Process of HIV-1 gp160 formation
NUP54	+	Nuclear pore protein	Nuclear import of PIC
PLS1	+	Actin-binding protein	Interact with HIVgp41
SDC2	+	Syndecan proteoglycan	Interact with HIV gp120, Tat and Matrix
TJP1	+	Tight junction proteins	gp120-mediated tight junction disruption
UBE2D1	+	Ubiquitin enzyme	Ubiquitination of HIV-1 Tat
FCAR	−	Immunoglobulin	The phenotype and function of monocytes
MKNK2	−	Protein kinases (CAMK)	MEK2-ERK pathway
ARHGEF1	−	Rho GTPases	Binds to HIV gp41
ESR2	−	Estrogen receptor	HIV gp120-induced cell death
EEF1D	−	Translation elongation	Enhance the translation of viral protein
GAA	−	Glucosidase	Processing of HIV-1 gp120
NFKBIB	−	NF-kappa-B inhibitor	NF-kappa-B pathway

In addition to the previously reported genes, RNA-seq analysis also identified many genes that were not previously reported to be functionally associated with HIV envelope proteins. Here, we listed the fold change and p value of the genes and their involved cellular pathways (Table 2). This table illustrates that genes encoding positive transcriptional regulators are upregulated, including TAF9, TAF9B, ZNF143, NPAT, CDK8, RNF2 and GTF2A2. In contrast, genes encoding negative transcriptional regulators were downregulated, including HDAC10, HDAC6, HDAC7, HSPBP1, DMAP1, RCOR2 and SMYD1. In addition, we noticed that the expression levels of a set of microRNAs were altered following the interaction between HIV vEnv and CD4/CXCR4 coreceptor. As shown in Table 2, the expression of several micro RNAs was downregulated, including that of miR181A2/B2, miR25, miR320A, miR342, miR423 and miR638. The RNA-seq data provided the evidence that vEnv-regulated cellular gene expression may have a great impact on HIV gene expression and/or viral replication, which deserves further investigation.

**Table 2:**
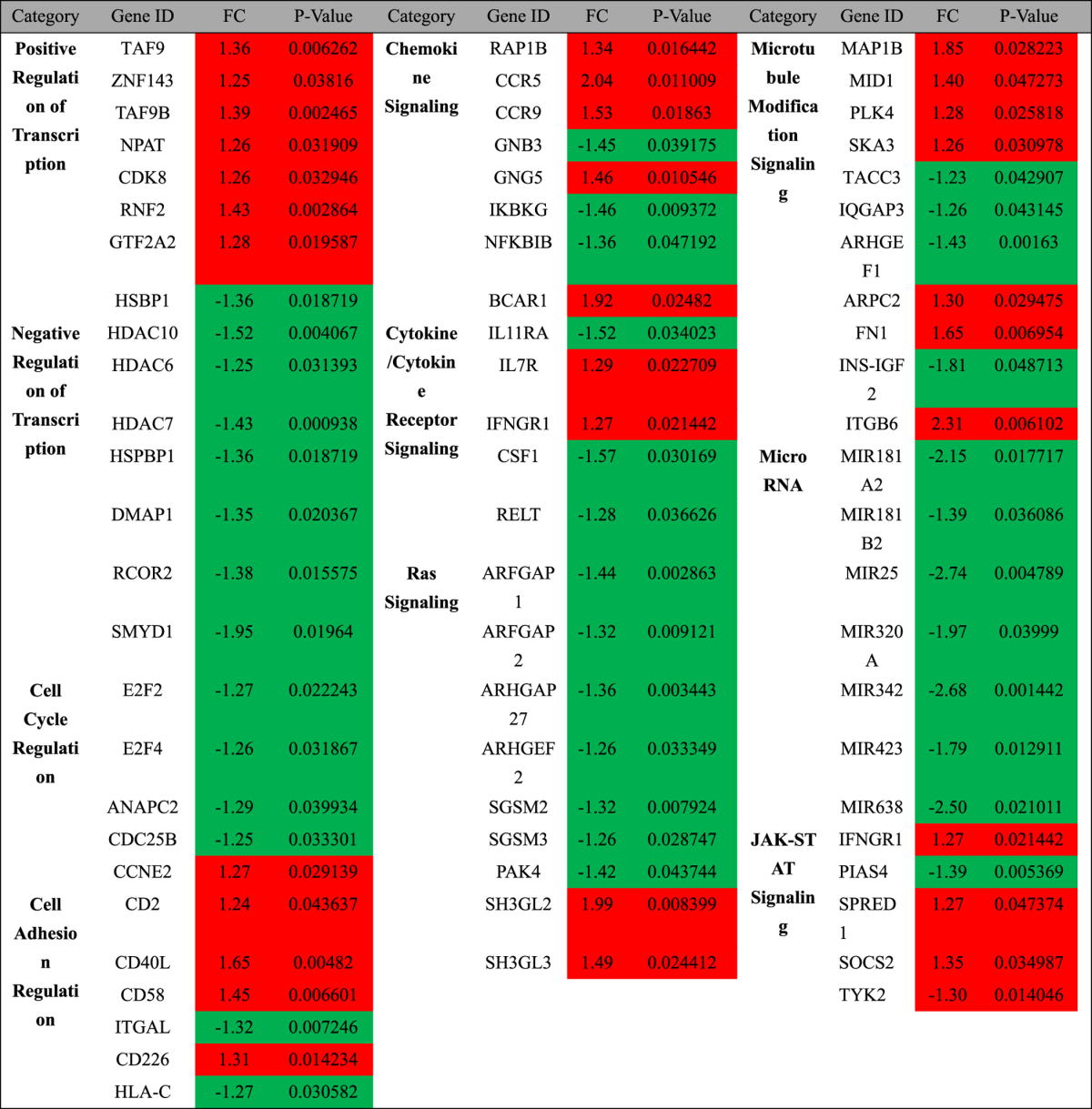
List of genes modulated by Env-VLP. Color coding is as follows: red indicates an increase and green a decrease in abundance compared to the untreated samples. P-value, p value of the genes’ expression in three duplicates; FC, fold change.

### vEnv regulates the expression of miR181A2 and P300/CBP-associated factors (PCAF) and increases HIV LTR-associated histone H3 acetylation

**Figure 5:**
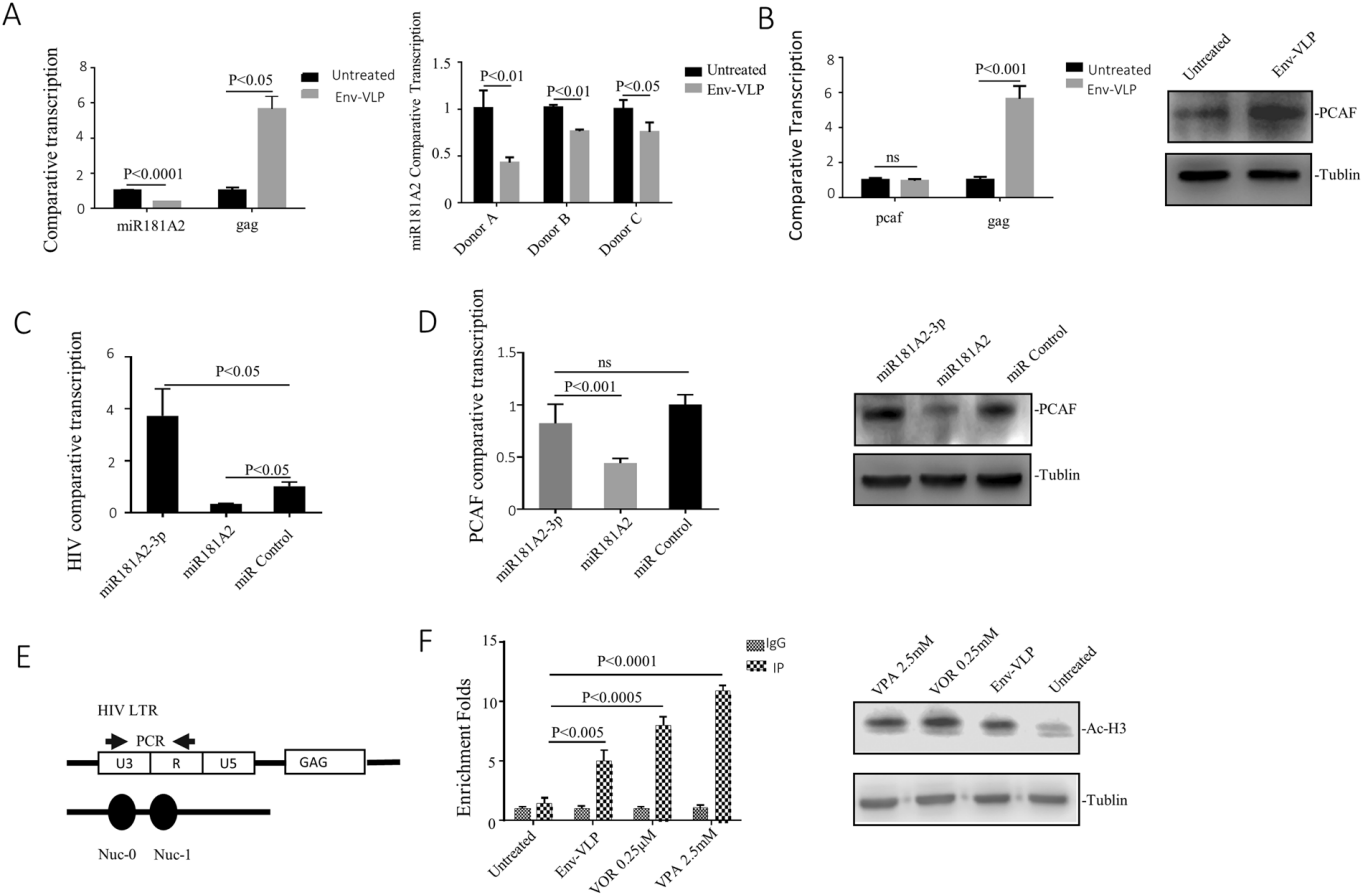
vEnv regulates the miR181A2/P300/CBP-associated factor (PCAF) expression, and increases HIV LTR histone H3 Acetylation. (**A**) The miR181A2 mRNA level was reduced in J-Lat 6.3 T cells treated with Env(X4)-VLP for 24 hrs, as compared to the untreated cells (n = 3) (left panel). The reduced miR181A2 mRNA levels in PBMCs from 3 donors treated with Env-VLP, as compared to the untreated cells (n = 2) (right panel). (**B**) The PCAF mRNA (left panel) and protein levels (right panel) in J-Lat 6.3 T cells treated with Env-VLP or untreated (n = 3). (**C**) The HIV gag transcription levels in J-Lat 6.3 T cells overexpressed with miR181A2 or miR181A2-3p (inhibitor), as compared to the cells transduced with empty lentiviral vectors (n = 3). J-Lat 6.3 T cells were transduced with lentiviral vectors encoding miR181A2, miR181A2-3p or transduced with empty vector for 24 hrs and kept on culture for another 72 hrs. Then, cells were collected and the HIV comparative transcription (gag/GAPDH) was measured by RT-PCR. (**D**) The PCAF mRNA (left panel) and protein levels (right panel) in miR181A2 or miR181A2-3p overexpressing J-Lat 6.3 T cells (n = 3). (**E**) The schematic diagram of the positions of the nucleosomes bound to the HIV-1 LTR and the location of the primers used for the real-time PCR in the ChIP assay. (**F**) The detection of histone H3 acetylation ratio in HIV LTR -109- + 82 by CHIP (n = 3). J-Lat 6.3 T cells were treated with Env(X4)-VLPs for 24 hrs. In parallel, cells treated with histone deacetylase inhibitor VOR or VPA were included as positive controls and the untreated cells act as a negative control. After treatment, cells were lysed and analyzed by CHIP assay (left panel). Also, the global histone H3 acetylation in J-Lat 6.3 T cells treated with Env(X4)-VLP, VOR, VPA or untreated were detected by western blot with anti-histone H3 acetylated antibody. Data are mean and sd. Ns, not significant p > 0.05. (Two-tailed unpaired t-test).

The above RNA-seq analysis revealed that the expression of miR181A2/B2 was downregulated by vEnv. Interestingly, a recent study reported that miR181a could downregulate PCAF expression by destabilizing PCAF mRNA^36^. PCAF is an acetyltransferase that uses the cofactor acetyl coenzyme A (AcCoA) to acetylate lysine 14 of histone 3, and overexpression of PCAF enhances histone H3 acetylation^37, 38^. We therefore speculate that HIV vEnv-mediated downregulation of miR181A2 would lead to increased expression of PCAF and facilitate histone H3 acetylation of HIV LTR, which in turn activates viral transcription. We first validated miR181A2 and PCAF expression in vEnv-treated J-Lat 6.3 T cells using individual RT-PCR assays and showed that the expression of miR181A2 was reduced by 60–70% in vEnv-treated cells compared to mock-treated cells (Fig. 5A left panel). The expression of PCAF was upregulated (Fig. 5B right panel) but without affecting the level of PCAF mRNA (Fig. 5B left panel). We also confirmed miR181A2 downregulation in PBMCs from three healthy donors upon vEnv treatment using RT-PCR (Fig. 5A right panel). We also evaluated the effect of miR181A2 on HIV transcription by overexpressing the miR181A2 or an miR181A2 inhibitor (miR181A2-3p^36^) in J-Lat 6.3 T cells using a lentiviral vector technique. After four days of introducing miR181A2 or miR181A2 inhibitors, HIV Gag mRNA expression was measured using RT-PCR. The results showed that the overexpression of miR181A2 led to a reduced HIV transcription, while the overexpression of miR181A2 inhibitor significantly increased HIV transcription (Fig. 5C).

To further address the relationship between miR181A2 and the expression of PCAF, we investigated the levels of PCAF in miR181A2 or miR181A2 inhibitor-overexpressed J-Lat 6.3 T cells by RT-PCR and western blotting. Interestingly, the overexpression of miR181A2 significantly downregulated PCAF mRNA and protein levels (Fig. 5D, left panel), while miR181A2 inhibitor efficiently increased the synthesis of PCAF protein but did not significantly change the PCAF mRNA level (Fig. 5D, right panel). These data suggest that the miR181A2/PCAF pathway is involved in the action of vEnv on viral transcription.

As PCAF plays a direct role in transcriptional regulation by enhancing histone H3 acetylation, it is necessary to investigate whether HIV LTR-associated H3 acetylation level changes upon stimulation with HIV vEnv. The J-Lat 6.3 T cells were treated with Env-VLP for 24 hours, followed by CHIP with anti-acetylated-histone H3 antibody and RT-PCR with primers located within the nucleosome-1 (nuc-1) of the HIV-1 LTR promoter (Fig. 5E). In parallel, cells treated with the histone deacetylase inhibitor vorinostat (VOR)^39^ or valproic acid (VPA)^40, 41^ were included as positive controls. The results showed that Env(X4)-VLP treatment was able to facilitate histone H3 acetylation in the 5′ LTR region (5-fold) compared to the untreated control, while VPA and VOR induced 11-fold and 8-fold acetylation, respectively (Fig. 5F, left panel). We further explored whether this enhanced acetylation is HIV LTR-specific. The level of histone H3 acetylation in J-Lat 6.3 T cells treated with Env-VLP, VOR or VPA was measured by western blotting using an anti-acetylated-histone H3 antibody (Fig. 5F, right panel). The scanning analysis revealed that global H3 acetylation in Env-VLP treated cells was approximately 1.79-fold higher than in mock-treated cells, which was not as high as that in cells treated with VOR (2.59-fold) or VPA (2.38-fold). All these observations provide evidence that miR181A2/PCAF/H3 acetylation is one cellular pathway manipulated by vEnv to activate HIV transcription.

### vEnv downregulates histone deacetylase 10 (HDAC10), which affects the infectivity of HIV progeny viruses

**Figure 6:**
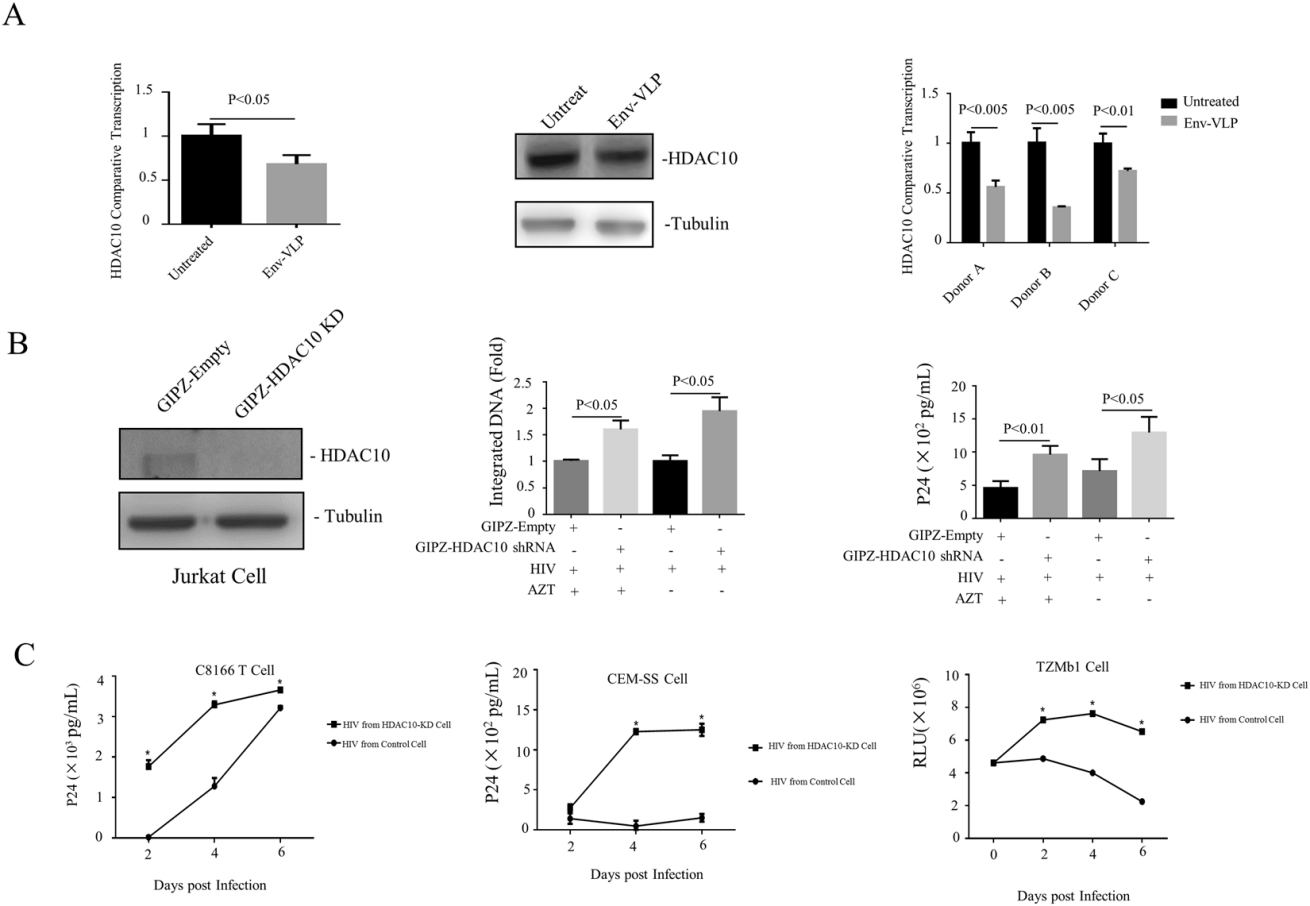
vEnv downregulates HDAC10 and enhances the infectivity of progeny viruses. (**A**) The HDAC10 levels in J-Lat 6.3 T cells (left panel) (n = 3) and PBMCs from 3 donors treated with Env(X4)-VLP (right panel) (n = 3) were measured by RT-PCR. Also, HDAC10 protein expressing levels in J-Lat 6.3 T cells treated with Env(X4)-VLP were also detected by western blot with anti-HDAC10 antibody (middle panel). (**B**) HDAC10 knock-down (KD) enhances HIV viral infection and/or replication. First, the shRNA-mediated HDAC10-KD in Jurkat T cells was checked by detecting HDAC10 expression with antiHDAC10 antibody (left panel). Then, both HDAC10-KD and the control Jurkat T cells were infected by HIV virus (N119) for 12 hrs. Then the infected cells were cultured in the presence or absence of AZT. After 72 hrs, the HIV integrated DNA was quantified by Alu-LTR- nested PCR procedure as described in Methods Section (middle panel), and HIVp24 level in the supernatant was detected by anti-p24 ELISA (right panel). (**C**) The progeny viruses from HIV infected HDAC10-KD Jurkat cells were more infectious than that from the control Jurkat cells. Equal amounts of progeny viruses (normalized by amounts of p24 levels) produced either from HDAC10-KD or the control Jurkat T cells were used to infect C8166 T cells, CEM-SS T cells, or TZMB1 cells. At different time intervals, the HIV Gag p24 levels in supernatant from infected C8166 T cells (left panel) and CEM-SS T cells (middle panel) were qualified by anti-p24 ELISA. Also, the HIV LTR-driving luciferase activity in TZMb1 cells infected with progeny virus from HDAC10-KD or control cells (n = 2) were detected by luciferase assay. Data are mean and sd. Ns, not significant, p > 0.05; *p < 0.05.(Two-tailed unpaired t-test; Multiple-t test; Correction for multiple comparison used the Holm-sidak method).

Another finding in our RNA-seq analysis was the vEnv-mediated downregulation of class IIb histone deacetylases (HDACs), including HDAC6 and HDAC10 (Table 2). HDAC6 has been documented for its inhibitory effect on cell fusion and entry of HIV through inhibition of the acetylation status of cortical tubulin^42^. It also regulates HIV Tat transactivation activity by binding to Tat and altering Tat acetylation at amino acid Lys-28^43^. However, the impact of HDAC10 during HIV infection and replication is still not known. We therefore investigated the effect of vEnv-mediated HDAC10 down-regulation on HIV replication. First, we validated the downregulation of HDAC10 in J-Lat 6.3 T cells upon treatment with Env(X4)-VLP by RT-PCR and western blotting. The results showed that in Env-VLP-treated cells, the expression of HDAC10 was reduced to 60–70% in mRNA level and to 60–70% in protein level (Fig. 6A left and middle panels). We also confirmed the down-regulation of HDAC10 in PBMCs from individual donors upon Env(X4)-VLP treatment (Fig. 6A right panel).

To further test whether the altered expression of HDAC10 could influence HIV production and/or infectivity, we first generated HDAC10 knock-down Jurkat T cells or control cells with GIPZ-HDAC10 shRNA or GIPZ-empty lenti-viral particles. After the HDAC10 knock-down efficiency was confirmed in Jurkat T cells (Fig. 6B left panel), the knock-down Jurkat T cells and control cells were infected with HIV virus (N119) for 12 hrs, and then the infected cells were cultured in the presence or absence of AZT. The goal of AZT treatment is to restrict HIV for only one round of replication. After 72 hrs, the HIVp24 level in the supernatant was detected by anti-p24 ELISA, and viral integrated DNA was detected by real time-PCR. The results showed that both viral integrated DNA level and the amount of produced p24 in the supernatant from the HDAC10-KD cells was 1.5- to 2-fold higher than in control cells, regardless of the presence of AZT (Fig. 6B middle and right panels). These data indicated that the reduced HDAC10 expression level facilitated the early steps towards to the establishment of HIV infection. Furthermore, when equal amounts of progeny viruses derived from the HDAC10-KD Jurkat T cells and the control T cells were used to infect the C8166 T cells, CEM-SS T cells and TZMb1 cells, we found that viruses produced from the HDAC10-KD Jurkat T cells mediated significantly higher levels of infection than the control Jurkat T cells (Fig. 6C), indicating that the infectivity of progeny viruses produced from the HDAC10-KD Jurkat T cells were significantly enhanced.

## Discussion

During HIV infection, it is known that a large proportion of produced progeny viral particles are defective, primarily due to error-prone viral reverse transcription^1, 2, 44^. Although numerous studies have shown that these defective particles, especially the envelope glycoprotein present on these viral particles, significantly contribute to HIV pathogenesis *in vivo* ^1, 45^, their roles and the mechanisms by which these virus-associated Env (vEnv) affect viral expression and late-stage viral replication remain elusive. In this study, we specifically investigated the effect of envelope glycoprotein present on HIV noninfectious viral particles on both HIV-LTR-driven transcription and host transcription. Our study demonstrated for the first time that noninfectious virus particles are able to stimulate HIV transcription in virus-infected cells, and the virion-associated envelope glycoprotein (vEnv) is the key factor for this transcription activation. We have observed this phenotype in various HIV-infected cells, including the HIV-infected J-Lat 6.3 T cells, HIV-infected resting PBMCs and resting PBMCs isolated from virologically suppressed HIV-infected patients. Furthermore, our transcriptome analysis revealed that treatment with vEnv modulates the expression of multiple genes that are involved in various cellular pathways, and contribute to HIV replication. Among these, our study showed that the vEnv-induced downregulation of miR181A2 and HDAC10 is functionally associated with vEnv-stimulated viral transcription and/or an enhanced infectivity of the produced progeny virus. All of these findings provide evidences for the important role of vEnv in facilitating HIV expression, replication and contributions to HIV pathogenicity.

The binding of gp120 with T cell receptor CD4 and coreceptors (CCR5 or CXCR4) is a necessary step for HIV entry into CD4 + T lymphocytes, monocytes and macrophages^13–15^. Previous studies have shown that the interaction between recombinant gp120 and CD4/coreceptors (CCR5 or CXCR4) enhances the early stage of viral infection. For instance, this interaction activates several pathways, including Tyrosine phosphorylation of Pyk2, Rho-GTPase, RhoA-ROCK, and ERM-Moesin, which induce cellular actin rearrangement and subsequently facilitate viral infection^46–49^. In this study, we found the interaction between gp120 and CD4/coreceptors also benefits the late stage of viral infection. Our data provided new evidence that vEnv is able to activate HIV LTR-driven transcription in HIV LTR-integrated cells through the interaction of HIV vEnv glycoprotein with CD4/coreceptors. Moreover, consistent with previous results that heat-inactivated virus can stimulate viral expression in HIV infected cells^50^, we further demonstrated that vEnv on the surface of inactivated virus was able to activate viral transcription in HIV latently-infected cells. These observations suggest that the presence of HIV-defective viral particles *in vivo* may be able to stimulate HIV-infected resting T cells and that at least some of them become productive HIV infected cells that contribute to a higher viral load and facilitate HIV dissemination.

Accumulated evidence suggests that HIV replication may occur in resting CD4 + T cells residing in the lymphoid tissue from HIV-1 infected individuals, either early in disease or after long-term ART^51, 52^. A recent study showed that HIV-infected resting CD4 + T cells can produce HIV Gag protein without spreading infection^53^. Thus, the observation of low-level productive infection in the presence of ART and a rapid rebound of viral replication after ART interruption suggest that undefined factors, including viral factors, are able to stimulate viral expression and replication *in vivo*, especially in human lymphoid tissue. These factors result in more infected CD4^+^ cells and the establishment an/or maintenance of latent viral reservoirs. Combined with previous studies revealing that vEnv may exist in the lymphoid tissue of HARRT-treated patients^51^, our observation may imply that HIV vEnv in lymphoid tissue may be a main factor that co-stimulates viral transcription and causes viral rebound. However, once HIV-infected resting PBMCs were stimulated by PHA or anti-CD3/CD28, no further effect was observed in the presence of vEnv. This is expected, since PHA or anti-CD3/CD28 treatment induces a strong activation status for T cells, enhancing viral transcription and therefore masking the effect of vEnv.

In patient serum, gp120 can be present in various forms. One is associated with viral envelope glycoprotein (gp41), which is present on the surface of the virus and/or on the infected cells as a trimer^10, 11, 54^. Another is the shed gp120, which is present in serum in a soluble form (soluble gp120)^55^. Whether they share the same stimulating activity on HIV transcription merits additional investigation. Although studies by Claudia in 2006 indicated that recombinant gp120 also activates NFAT1 and induces NFAT1 translocation from cytosol into nucleus in resting CD4 + T cells isolated from HIV-infected individuals^56^, we found that virion-associated gp120 (vEnv), but not soluble gp120, had a profound effect on HIV transcription (Fig. 2). It is known that the mature Env complex is composed of three gp120 exterior subunits and three gp41 transmembrane subunits. In addition, MA trimerization is required to form a lattice capable of accommodating the long cytoplasmic tail of HIV-1 Env^57^. Thus, it is reasonable that Env glycoprotein associated with virus-like particles forms the biologically active complex that may play a major role in HIV-1 pathogenesis. The reason for the minimal activity of shed gp120 in our study may be that it is not in a functional conformation. However, we did not further test whether the shed gp120 could induce other biological activities.

Next, we asked how HIV transcription could be stimulated by vEnv. Numerous previous studies have shown that the binding of gp120 to CD4/coreceptors can modulate or activate multiple cellular pathways^7^. To further elucidate the relationship with HIV transcription, we performed RNA-seq analysis. The results revealed that the host genes modulated by vEnv are associated with Ras signaling, cell adhesion, cell cycle regulation, actin skeleton organization and transcription regulation. In addition to the genes reported previously, we also found a number of genes, including HDAC6, HDAC10 and several micro RNAs, which are responsive to vEnv stimulation (listed in Table 2). Among these genes, the microRNA miR181A2 was significantly downregulated by vEnv. In HIV latently infected J-Lat 6.3 T cells, we found that the downregulation of miR181A2 enhanced the expression of PCAF and activated HIV LTR-associated histone H3 acetylation, potentially contributing to the activation of HIV transcription (Fig. 5). Hence, it can be speculated that HIV is able to use its Env glycoprotein to stimulate its transcription by down-regulating miR181A2 and enhancing LTR histone acetylation, thus providing one possible mechanism for rapid viral rebound after HARRT treatment ceases. In HIV latently infected cells, HIV transcription is absent or rare due to low expression of Tat and high deacetylation in the HIV LTR region. Once HARRT treatment ceases, the Env glycoprotein present on small amounts of viruses can interact with the receptor/coreceptors on HIV-infected resting T cells and modulate multiple cellular signaling factors including the down-regulation of cellular miR181A2 and facilitation of HIV LTR acetylation. These in turn may contribute to the rebound viremia. Thus, regulation of miR181A2 appears to be a natural host machinery targeted by HIV, and the down-regulation of miR181A2 may be an attractive strategy to induce HIV reactivation in chronically infected individuals to eliminate HIV reservoirs.

Another interesting finding in this study is that in addition to acting on viral expression, vEnv stimulation could also augment the infectivity of the newly produced progeny virus. It is evident that vEnv treatment led to downregulation of cellular HDAC10 and HDAC6 expression (Table 2 and Fig. 6A), and downregulation of HDAC10 by shRNA knock-down significantly enhanced HIV viral infection and/or replication, especially through facilitation of progeny virion infectivity (Fig. 6B,C). Although the mechanisms are still required to be elucidated, some previous studies revealed that HDAC10 can physically interact with HSP70 to affect its acetylation status, which may affect HSP70 activity^58^. The induction of HSP70 inhibits HIV-1 Vif-mediated degradation of APOBEC3G, which interrupts HIV infectivity by introducing dC-to-dU mutations in the minus viral DNA strand during reverse transcription^59–62^. Therefore, it is possible that HDAC10 downregulation enhances progeny virion infectivity through enhanced Vif-mediated degradation of APOBEC3G. It could also be possible that HDAC10 downregulation could affect viral protein integrase acetylation status, as in the case of p300^63^, which may enhance integrase catalytic activity and viral infectivity. Another question of how significant role it could play during HIV infection *in vivo* still remains to be investigated. Since in Env-VLP-treated Jurkat cells, the expression of HDAC10 was only reduced to 60–70% (Fig. 6A left and middle panels), and in the Env-VLP-treated PBMCs, HDAC10 also was downregulated at various levels (Fig. 6A, right panel). It appears that HDAC10 downregulation is one of multiple pathways manipulated by vEnv to facilitate HIV infection. Successful identifying these mechanisms to be involved in vEnv-mediated facilitation of viral infectivity merits further investigation and may open new avenues to fight HIV infection.

Overall, this study for the first time provides evidences for the important role of vEnv in modulating cellular environments in the HIV infected cells and for stimulating HIV expression and infectivity of produced progeny viruses. It should be noted that, although both × 4- and R5-tropic vEnv were able to stimulate HIV LTR-driving expression in TZMb1 cells (Figs 1 and 2), our study was mainly focused on the underlying mechanism of Env (X4) in viral transcription activation and progeny virus infectivity in HIV infected CD4 + T cells. We still do not know whether Env(R5)-activated signaling pathways could be different from that by vEnv(X4). Further studies will continue to characterize the role of Env (R5) during viral infection in macrophages and investigate its effect on cellular signaling pathways. Also, the findings of the presence of vEnv is able to stimulate multiple cellular signaling pathways in CD4 + T cells in this study may have implications for a continuing activation of CD4 + T cells in the late stage of chronic HIV infection, which lead to the exhaustion of these T cells and their apoptosis as described previously^64–67^. So, we believe that more detailed investigations to elucidate the biological relevance of vEnv during HIV infection *in vivo* and better understanding of the involved mechanisms may provide new avenues for designing novel strategies against HIV infection.

## Materials and Methods

### Plasmid, chemicals and antibodies

HIV-1 plasmid carrying a secreted Gaussia luciferase (Gluc) gene (ΔRT/ΔEnv/Gluciferase), HIV-packaging plasmids pCMVΔ8.2, or CMVinGag-Pol, HIV-1 envelope glycoprotein plasmids pLET-Lai (X4-tropic) and pLET-JRFL (R5-tropic), CMV-VSV-G, CMV-HA and CMV-ZGP plasmids (pCAGGS-ZEBOV-GP) were described previously^22, 68–70^. Lentiviral-vector encoding hs-miR-181a-2-3p miRNA, hsa-miR-181a-2 and miR control plasmids were purchased from Applied Biological Materials Inc (Richmond, BC). GIPZ-HDAC10 shRNA lentiviral plasmid and GIPZ-Empty control plasmid were purchased from Open Biosystem. Bicyclam JM-2987, TAK 779, vorinostat (VOR), recombinant HIV-1 gp120 (CM) and HIV-1 gp120 (Bal) were obtained from the NIH AIDS Research and Reagent Program. Valproic acid (VPA) and phytohemagglutinin (PHA) were purchased from Sigma and Roche.

The antibodies used for Western blotting (WB) were as follows: a rabbit anti-green fluorescent protein (anti-GFP) polyclonal antibody (Molecular Probes), a mouse anti-β-tubulin antibody (Sigma), mouse anti-PCAF (Santa Cruz), mouse anti-HDAC10 (Santa Cruz) and rabbit polyclonal antibodies against avian influenza NA protein (Cedarlane Lab, Ontario). HIV-1 gp120 monoclonal antibody (ID6) and human HIV antibody NIH45-46G54W were obtained from the NIH AIDS Research and Reagent Program. Mouse monoclonal antibody (2G4) against EBOV glycoprotein was described previously^71^. HRP-conjugated donkey anti-rabbit IgG and sheep anti-mouse IgG were purchased from Amersham Biosciences (Mississauga, Ontario).

### Primary cell isolation and cell culture

Human embryonic kidney 293 T, TZM-b1, J-Lat 6.3 and C8166 T cells were cultured in Dulbecco’s modified Eagle’s medium (DMEM) or RPMI-1640 medium, each supplemented with 10% fetal bovine serum (FBS), 100 unit/ml penicillin and 100 μg/ml streptomycin. Peripheral blood mononuclear cells (PBMCs) were isolated from the blood of healthy adult volunteers or HIV latent patients by sedimentation in Ficoll-Hypaque (Sigma-Aldrich St Louis, MO). CD4 + T lymphocytes were isolated from peripheral blood mononuclear cells by negative selection with an EasyStep Human CD4 + CD25 + T Cell Isolation Kit (Stemcell Technologies, Vancouver, Canada). Purified resting CD4 + T cells and PBMCs were cultured in RPMI-1640 medium supplemented with 10% fetal bovine serum, 100 unit/ml penicillin and 100 μg/ml streptomycin and 10 μg/ml IL-2. All HIV-related analyses in isolated PBMCs from HIV latent patients were conducted in accordance with our internal ethics committee and were approved by the Ethics Committee from University of Manitoba. Written informed consent was obtained from all subjects.

### Virus and virus-like particle (VLP) preparation and infection

Nevirapine-resistant × 4 tropic HIV-1 (N119, Cat1392) was obtained from the NIH AIDS Research and Reference Reagent Program. HIV-1 Env-VLPs were generated by co-transfecting 293 T cells with Gag-pol expressor pCMVΔ8.2, and pLET-Lai (X4-tropic) or pLET-JRFL (R5-tropic). HA-NA-M2-VLP, Ebola-Env-VLP and VSV(G)-VLP were generated by co-transfecting 293 T cells with pCMVΔ8.2 and CMV-VSV-G, CMV-HA or CMV-ZGP, respectively. Lentiviral particles expressing MiR181A2, MiR181A2-3p were produced by co-transfecting 293 T cells with VSV-G expression plasmid, pCMVΔ8.2 and lentiviral vector HSA-MiR-181A2-3p / HSA-MiR-181A2/miR Control, respectively. One cycle of replicating virus (Gluc + ) was generated by cotransfecting 293 T cells with HIV ΔRT/ΔEnv/Gluciferase plasmid, CMVinGag-Pol, and VSV-G plasmid. After 48 hours of transfection, all supernatants were collected and spun at 3000 rpm for 30 minutes to clear debris, and the viruses or VLPs were concentrated by ultracentrifugation at 35000 rpm for 90 minutes. Concentrated virus or VLP stocks were quantified for Gagp24 levels using an anti-p24 ELISA kit. Virus inactivation: HIV virus was treated with indicated concentrations of aldrithiol-2 (AT-2) for 1 hr at 37 °C and filtered with centrifuge filters (Amicon Ultra-4) to remove AT-2 for further analysis^72^.

For PHA-stimulated PBMCs, isolated PBMCs were first cultured in RPMI 1640 containing IL-2 (10 μg/mL) and PHA (3 µg/ml) for three days. Then, 1 × 10^6^ stimulated PMBCs were infected with 10 ng (p24 gag) of VSV-G pseudotyped one cycle Gluc + HIV-1 for two hrs, washed and cultured in RPMI medium without PHA. Forty-eight hours later, the infected cells were prepared for further experiments. For CD3/CD28-stimulated PBMCs, isolated cells were cultured with anti CD3/CD28 (25 μl/1 million cells) for two days followed by infection with 1 ng (p24) of HIV-1 (N119) for two hrs. After additional culturing for 48 hrs, the infected cells were ready for further experiments. Resting primary CD4 + T lymphocytes isolated from peripheral blood mononuclear cells of healthy donors were cultured in RPMI supplemented with 10% FBS and IL-2 (10 µ/ml) for 2 days, and 1 × 10^6^ cells were then infected with 10 ng (p24) of HIV-1 (N119) by spinoculation at 1400 rpm for 2 hrs at room temperature. After overnight incubation, cells were washed and cultured in RPMI containing IL-2 for three days to establish latent infection.

The miR181A2 and miR181A2-3p–VLPs were used to transduce J-Lat 6.3 T cells, and the cells were washed after 24 hrs of transduction. After 96 hrs, the cells were collected and used for further analysis. To generate HDAC-knock-down cells, the Jurkat T cells were transduce with The GIPZ-Empty and GIPZ-HDAC10 shRNA lentiviral particles. After 96 hrs, the cells were selected with 2 μg/ml puromycin for seven days and collected for further analysis.

### Env-VLP, gp120, and shed gp120 treatment of cells

TZM-b1 cells in each well of a 24-well plate were treated with varying concentrations of HIV-1 Env-VLP (1–10 ng p24Gag), VSV, H5N1 or Ebola envelope pseudotype-VLPs (4 ng p24Gag) or recombinant HIV-1 gp120 (0.5–2 µg), HIV-1 Bal gp120 (40–80 ng) for the indicated time points, ranging from 4 to 48 hours. To enhance the binding of envelope to cell receptors, TZM-b1 cells were spinoculated at 1200 rpm for 30 min after adding VLPs. To assess the specific effect of gp120-CD4/coreceptors interactions on the action of Env-VLP, TZM-b1 cells were first incubated with varying concentrations of HIV-1 neutralizing antibody NIH45-46G54W (targeted to the CD4/gp120 interaction region), TAK-779 or Bicyclam JM-2978 for two hours following 24 hrs of HIV Env(X4/R5)-VLP treatment. The treated cells were collected and subjected to RT-PCR or luciferase activity assays as described below.

For assessment of the effect of the shed HIV gp120, and the recombinant gp120 on HIV LTR-transcription, was produced from HIV-1-transfected 293 T cells or infected C8166 cells. Briefly, 293 T cells were transfected with PNL4.3 plasmid for 48 hours, while C8166 T cells were infected with HIV PNL 4.3 for 48 to 72 hours. The supernatants from transfected 293 T cells or infected C8166 cells were collected following ultracentrifugation to separate the shed gp120 from pelleted viruses. The shed gp120 in the supernatants was further concentrated using a Vivaspin 6 centrifugal concentrator (100MWCO PES). Various volumes of shed gp120 were added to TZM-b1 cells for 24 hours, and the luciferase activity of cells was measured by luciferase assay (Promega).

J-Lat 6.3, infected stimulated or resting PBMC cells and PBMCs isolated from patients were treated with Env-VLP at a concentration of 6 μg of p24 per 1 × 10^6^ cells, spinoculated at 1200 rpm for 30 minutes and cultured for 24 or 48 hours for further analysis.

PBMCs were isolated from various healthy individuals, and CD4 + T cells were isolated from PBMCs using a Human CD4 + T cell enrichment kit (Stem Cells). The freshly purified CD4 + T cells were treated with Env-VLP for 24 hours and washed with PBS twice for further analysis.

### RT-PCR and data analysis

Various Env-VLP-treated cells were collected and washed with PBS twice. Total RNA was isolated from cells using a High Pure RNA Isolation Kit (Roche) and reverse-transcribed into cDNA with M-MLV reverse transcriptase (Promega). The relative expression levels (comparative transcription) of target mRNA was detected by RT-PCR analysis and normalized using the household gene GAPDH. RT-PCR reactions were performed by a two-step reaction in a 20 µl total volume using a LightCycler® 480 SYBR Green I Master kit (Roche), and in a thermocycler (Stratagene MX3000) with the following protocol: one cycle of 95 °C for 10 minutes; and 40 cycles of 95 °C for 10 seconds, 60 °C for 30 seconds, and 72 °C for 30 seconds. The data were organized using the Prism program, and the p value was calculated using unpaired t tests. The primer sequences are as follows: 5′-Gag: ATCAAGCAGCCATGCAAATG; 3′-Gag: CTGAAGGGTACTAGTAGTTCC; 5′-Gfp: GGTGGTGCAGATGAACTTCA; 3′-Gfp: AACCACTACCTGAGCAC; 5′-Gapdh: TGGGTGTGAACCATGAGAAG; 3′-Gapdh: ATGGACTGTGGTCATGAGTC; 5′-PCAF: TGCTGTCAGTATTTTAACACCC; 3’-PCAF: GCACTAAACTGGAATCCCAAG; 5′- miR181A2: TCAGAGGACTCCAAGGAACATT; 3′-miR181A2: GCTAACGGTCAG TGGTTTTTTC; 5′-HDAC10: ATGTGGCTGTTCGGAGAGGC; 3′-HDAC10: CTGCACTCCTGGCTGCAATG.

### Luciferase assay and Western blotting

After being treated with Env-VLPs or other reagents, TZMb1 cells were collected and washed with PBS twice. The cell pellet was lysed with lysis buffer (Promega) and the luciferase activity in the supernatant was detected using a GLOMAX Luminometer (Promega) and normalized using the total protein concentration.

To detect viral envelope proteins incorporated in the VLPs, 4 ng (p24 Gag) of various purified VLPs were lysed in RIPA buffer and directly loaded onto a 10% SDS-PAGE gel. The viral protein Gag-p24 and the envelope gp120 or other viral glycoproteins were detected using various specific antibodies. Recombinant HIV-1 gp120 and shed gp120 from transfected or infected cells was detected by loading serially diluted recombinant protein or concentrated supernatants onto a 10% SDS-PAGE gel following probing with anti-gp120 monoclonal antibodies. To detect the expression of PCAF, acetylated histone H3, GFP or HDAC10 proteins, cells were lysed in 1% NP-40, normalized by total protein concentration, loaded onto 10–12% SDS-PAGE and detected using corresponding antibodies.

### ChIP (chromatin immunoprecipitation)

According to the steps described previously^73^, different treated cells were collected, cross-linked with formaldehyde to a final concentration of 1% for 10 min at 37 °C and lysed on ice for 30 minutes. The cell debris was cleared by centrifugation at 13,000 rpm for 10 minutes. The supernatant was transferred to a new tube and sonicated for 15 times of 10-second bursts. Next, the histone H3 antibody or IgG (Millipore) was added to the lysis mixture, and the liquid was incubated with Protein A beads (Sigma) at 4 °C overnight. The beads were then washed and mixed with 10% Chelex*100 slurry (Sigma) and boiled for 10 minutes. The samples underwent proteinase K treatment, and the proteinase K was inactivated at 55 °C for 30 minutes. Last, the DNA was purified and prepared for further analysis. The copy of the LTR -109− + 82 region was detected by RT-PCR with the primer pair LTR -109− + 82 (F: TACAAGGGACTTTCCGCTGG) and LTR -109− + 82 (R: AGCTTTATTGAGGCTTAAGC), and the fold enrichment was calculated using 2^−(CtIP-CtIgG)^ ^74^.

### RNA-Seq: Total RNAseq library preparation and sequencing

Whole-genome sequencing was carried out at the next-generation sequencing (NGS) facility of the Manitoba Institute of Child Health (MICH) using the Illumina MiSeq platform. Briefly, J-Lat 6.3 T cells were treated with Env-VLP or untreated for 24 hrs, and total RNA was extracted from J-Lat 6.3 T cells using a Nucleospin kit (Macherey-Nagel, Germany) according to the protocol provided by the manufacturer. Total RNA was depleted of ribosomal RNA using the Epicentre Ribo-Zero Magnetic Kit, fragmented using RNaseIII and assessed for yield and size by the Agilent Bioanalyzer 2100. The fragmented RNA was hybridized and ligated to SOLiD adaptors, and reverse transcription was performed. The cDNA was size-selected to remove fragments smaller than 100 bp using Agencourt AMPure (Beckman Coulter) magnetic beads and subjected to limited PCR amplification. After purification, the resulting libraries were quantitated using Qubit and assessed again for size and quality using a Bioanalyzer. The libraries were pooled to be equimolar and were amplified on SOLiD sequencing beads using the EZ bead system (ABI). The beads were loaded into one lane of a SOLiD flowchip and sequenced on the SOLiD5500xl platform (ABI) using paired-end (50 × 35 bp) tags.

### Statistical analysis

Statistical analysis of Env-VLP functional assays, including the results of luciferase assays, comparative RT-PCR and p24 ELISA, were performed using the unpaired t test (considered significant at *P* ≤ 0.05) or multiple t test (with correction using the Holm-sidak method) using GraphPad Prism 6.01 software.

RNA-seq bioinformatics data analysis: A total of 201,127,972 pairs of SOLiD sequence reads were generated from the samples. After quality checking, 158,718,406 of these reads were mapped onto the human reference genome (hg19) using Lifescope v2.5.1 software (Life Technologies) with the 2-mismatch setting. The mapped reads were quantified against the gene features of the seq database. The gene expression values were normalized as RPKM (reads per kilobase per million reads). The bioconductor package edgeR, based on a Negative binomial model, was used to infer the differential expression gene (DEG) expression^75^.

## Electronic supplementary material

Supplementary Information

## References

[1] Finzi D, Plaeger SF, Dieffenbach CW (2006). Defective virus drives human immunodeficiency virus infection, persistence, and pathogenesis. Clinical and vaccine immunology.

[2] Thomas JA, Ott DE, Gorelick RJ (2007). Efficiency of human immunodeficiency virus type 1 postentry infection processes: evidence against disproportionate numbers of defective virions. Journal of virology.

[3] Bourinbaiar A (1994). The ratio of defective HIV-1 particles to replication-competent infectious virions. Acta virologica.

[4] Marozsan AJ (2004). Relationships between infectious titer, capsid protein levels, and reverse transcriptase activities of diverse human immunodeficiency virus type 1 isolates. Journal of virology.

[5] Aupeix K (1997). The significance of shed membrane particles during programmed cell death *in vitro*, and *in vivo*, in HIV-1 infection. Journal of Clinical Investigation.

[6] Kameoka M (1997). Protease-defective, gp120-containing human immunodeficiency virus type 1 particles induce apoptosis more efficiently than does wild-type virus or recombinant gp120 protein in healthy donor-derived peripheral blood T cells. Journal of clinical microbiology.

[7] Wu, Y. & Yoder, A. Chemokine coreceptor signaling in HIV-1 infection and pathogenesis. (2009). 10.1371/journal.ppat.1000520 PMC2790611 20041213

[8] McKeating JA, Willey RL (1989). Structure and function of the HIV envelope. Aids.

[9] Caffrey M (2011). HIV envelope: challenges and opportunities for development of entry inhibitors. Trends in microbiology.

[10] Chertova E (2002). Envelope glycoprotein incorporation, not shedding of surface envelope glycoprotein (gp120/SU), is the primary determinant of SU content of purified human immunodeficiency virus type 1 and simian immunodeficiency virus. Journal of Virology.

[11] Zhu P (2006). Distribution and three-dimensional structure of AIDS virus envelope spikes. Nature.

[12] SATI’ENTAU, Q. I. Dissociation of Induced by Soluble CD4. (1990).

[13] Dragic T (1996). HIV-1 entry into CD4 sup + cells is mediated by the chemokine receptor CC-CKR-5. Nature.

[14] Chan DC, Kim PS (1998). HIV entry and its inhibition. Cell.

[15] Berger EA, Murphy PM, Farber JM (1999). Chemokine receptors as HIV-1 coreceptors: roles in viral entry, tropism, and disease. Annual review of immunology.

[16] Yoder A (2008). HIV envelope-CXCR4 signaling activates cofilin to overcome cortical actin restriction in resting CD4 T cells. Cell.

[17] Cicala C (2002). HIV envelope induces a cascade of cell signals in non-proliferating target cells that favor virus replication. Proceedings of the National Academy of Sciences.

[18] Del Cornò M (2014). HIV-1 gp120 activates the STAT3/interleukin-6 axis in primary human monocyte-derived dendritic cells. Journal of virology.

[19] Marschner S, Hünig T, Cambier JC, Finkel TH (2002). Ligation of human CD4 interferes with antigen-induced activation of primary T cells. Immunology letters.

[20] Zhu P (2003). Electron tomography analysis of envelope glycoprotein trimers on HIV and simian immunodeficiency virus virions. Proceedings of the National Academy of Sciences.

[21] Takeuchi Y, McClure MO, Pizzato M (2008). Identification of gammaretroviruses constitutively released from cell lines used for human immunodeficiency virus research. Journal of virology.

[22] Jayappa KD (2015). Human Immunodeficiency Virus Type 1 Employs the Cellular Dynein Light Chain 1 Protein for Reverse Transcription through Interaction with Its Integrase Protein. Journal of virology.

[23] Ao Z (2008). Characterization of a trypsin-dependent avian influenza H5N1-pseudotyped HIV vector system for high throughput screening of inhibitory molecules. Antiviral research.

[24] Zhang, X. *et al*. Characterization of the inhibitory effect of an extract of Prunella vulgaris on Ebola virus glycoprotein (GP)-mediated virus entry and infection. *Antiviral Research* (2016). 10.1016/j.antiviral.2016.01.001 PMC7113790 26778707

[25] Wu Y, Yoder A (2009). Chemokine coreceptor signaling in HIV-1 infection and pathogenesis. PLoS pathogens.

[26] Klein F (2012). HIV therapy by a combination of broadly neutralizing antibodies in humanized mice. Nature.

[27] Dragic T (2000). A binding pocket for a small molecule inhibitor of HIV-1 entry within the transmembrane helices of CCR5. Proceedings of the National Academy of Sciences.

[28] Hendrix CW (2000). Pharmacokinetics and safety of AMD-3100, a novel antagonist of the CXCR-4 chemokine receptor, in human volunteers. Antimicrobial agents and chemotherapy.

[29] Oh S-K (1992). Identification of HIV-1 envelope glycoprotein in the serum of AIDS and ARC patients. JAIDS Journal of Acquired Immune Deficiency Syndromes.

[30] Herbein G (1998). Apoptosis of CD8&plus; T cells is mediated by macrophages through interaction of HIV gp120 with chemokine receptor CXCR4. Nature.

[31] Ivey-Hoyle M (1991). Envelope glycoproteins from biologically diverse isolates of immunodeficiency viruses have widely different affinities for CD4. Proceedings of the National Academy of Sciences.

[32] Jordan A, Bisgrove D, Verdin E (2003). HIV reproducibly establishes a latent infection after acute infection of T cells *in vitro*. The EMBO journal.

[33] Yerly S (1999). Transmission of antiretroviral-drug-resistant HIV-1 variants. The Lancet.

[34] Buckheit RW (1994). Biological and biochemical anti-HIV activity of the benzothiadiazine class of nonnucleoside reverse transcriptase inhibitors. Antiviral research.

[35] Huang DW, Sherman BT, Lempicki RA (2009). Systematic and integrative analysis of large gene lists using DAVID bioinformatics resources. Nature protocols.

[36] Zhao J (2012). Downregulation of PCAF by miR-181a/b provides feedback regulation to TNF-α–induced transcription of proinflammatory genes in liver epithelial cells. The Journal of Immunology.

[37] Nagy Z, Tora L (2007). Distinct GCN5/PCAF-containing complexes function as co-activators and are involved in transcription factor and global histone acetylation. Oncogene.

[38] Schiltz RL (1999). Overlapping but distinct patterns of histone acetylation by the human coactivators p300 and PCAF within nucleosomal substrates. Journal of Biological Chemistry.

[39] Archin NM (2012). Administration of vorinostat disrupts HIV-1 latency in patients on antiretroviral therapy. Nature.

[40] Ylisastigui L, Archin NM, Lehrman G, Bosch RJ, Margolis DM (2004). Coaxing HIV-1 from resting CD4 T cells: histone deacetylase inhibition allows latent viral expression. Aids.

[41] Lehrman G (2005). Depletion of latent HIV-1 infection *in vivo*: a proof-of-concept study. The Lancet.

[42] Valenzuela-Fernandez A, Cabrero JR, Serrador JM, Sánchez-Madrid F (2008). HDAC6: a key regulator of cytoskeleton, cell migration and cell–cell interactions. Trends in cell biology.

[43] Huo L (2011). Regulation of Tat acetylation and transactivation activity by the microtubule-associated deacetylase HDAC6. Journal of Biological Chemistry.

[44] Fuller SD, Wilk T, Gowen BE, Kräusslich H-G, Vogt VM (1997). Cryo-electron microscopy reveals ordered domains in the immature HIV-1 particle. Current Biology.

[45] Ohki K (1991). Noninfectious doughnut-shaped human immunodeficiency virus type 1 can induce syncytia mediated by fusion of the particles with CD4-positive cells. JAIDS Journal of Acquired Immune Deficiency Syndromes.

[46] Davis CB (1997). Signal transduction due to HIV-1 envelope interactions with chemokine receptors CXCR4 or CCR5. The Journal of experimental medicine.

[47] Barrero-Villar M (2009). Moesin is required for HIV-1-induced CD4-CXCR4 interaction, F-actin redistribution, membrane fusion and viral infection in lymphocytes. Journal of cell science.

[48] Hodges A (2007). Activation of the lectin DC-SIGN induces an immature dendritic cell phenotype triggering Rho-GTPase activity required for HIV-1 replication. Nature immunology.

[49] Jiménez-Baranda S (2007). Filamin-A regulates actin-dependent clustering of HIV receptors. Nature Cell Biology.

[50] Briant L, Coudronniere N, Robert-Hebmann V, Benkirane M, Devaux C (1996). Binding of HIV-1 virions or gp120-anti-gp120 immune complexes to HIV-1-infected quiescent peripheral blood mononuclear cells reveals latent infection. Journal of Immunology.

[51] Santosuosso M, Righi E, Lindstrom V, Leblanc PR, Poznansky MC (2009). HIV-1 envelope protein gp120 is present at high concentrations in secondary lymphoid organs of individuals with chronic HIV-1 infection. Journal of Infectious Diseases.

[52] Fauci AS (1993). HIV infection is active and progressive in lymphoid tissue during the clinically latent stage of disease. Nature.

[53] Pace MJ (2012). Directly infected resting CD4 + T cells can produce HIV Gag without spreading infection in a model of HIV latency. PLoS Pathog.

[54] Julien J-P (2013). Crystal structure of a soluble cleaved HIV-1 envelope trimer. Science.

[55] Moore, J. P., McKeating, J. A., Weiss, R. A. & Sattentau, Q. J. Dissociation of gp120 from HIV-1 virions induced by soluble CD4. *Science* **250**, 1139-1142 (1990). 10.1126/science.2251501 2251501

[56] Cicala C (2006). HIV-1 gp120 induces NFAT nuclear translocation in resting CD4 + T-cells. Virology.

[57] Cosson P (1996). Direct interaction between the envelope and matrix proteins of HIV-1. The EMBO journal.

[58] Oehme I (2013). Histone deacetylase 10 promotes autophagy-mediated cell survival. Proceedings of the National Academy of Sciences.

[59] Zhang H (2003). The cytidine deaminase CEM15 induces hypermutation in newly synthesized HIV-1 DNA. Nature.

[60] Lecossier D, Bouchonnet F, Clavel F, Hance AJ (2003). Hypermutation of HIV-1 DNA in the absence of the Vif protein. Science.

[61] Mangeat B (2003). Broad antiretroviral defence by human APOBEC3G through lethal editing of nascent reverse transcripts. Nature.

[62] Sugiyama R (2013). Induction of heat-shock protein 70 by prostaglandin A 1 inhibits HIV-1 Vif-mediated degradation of APOBEC3G. Antiviral research.

[63] Allouch A, Cereseto A (2011). Identification of cellular factors binding to acetylated HIV-1 integrase. Amino acids.

[64] Cicala C (2000). HIV-1 envelope induces activation of caspase-3 and cleavage of focal adhesion kinase in primary human CD4 + T cells. Proceedings of the National Academy of Sciences.

[65] Ohnimus H, Heinkelein M, Jassoy C (1997). Apoptotic cell death upon contact of CD4 + T lymphocytes with HIV glycoprotein-expressing cells is mediated by caspases but bypasses CD95 (Fas/Apo-1) and TNF receptor 1. The Journal of Immunology.

[66] Ahr B, Robert-Hebmann V, Devaux C, Biard-Piechaczyk M (2004). Apoptosis of uninfected cells induced by HIV envelope glycoproteins. Retrovirology.

[67] Doitsh G (2010). Abortive HIV infection mediates CD4 T cell depletion and inflammation in human lymphoid tissue. Cell.

[68] Yao X-J (1998). Vpr stimulates viral expression and induces cell killing in human immunodeficiency virus type 1-infected dividing Jurkat T cells. Journal of virology.

[69] Li S (1999). Recombinant influenza A virus vaccines for the pathogenic human A/Hong Kong/97 (H5N1) viruses. Journal of Infectious Diseases.

[70] Wahl-Jensen V (2005). Role of Ebola virus secreted glycoproteins and virus-like particles in activation of human macrophages. Journal of virology.

[71] Qiu X (2011). Characterization of Zaire ebolavirus glycoprotein-specific monoclonal antibodies. Clinical immunology.

[72] Rossio J (1998). Inactivation of human immunodeficiency virus type 1 infectivity with preservation of conformational and functional integrity of virion surface proteins. Journal of virology.

[73] Nelson JD, Denisenko O, Bomsztyk K (2006). Protocol for the fast chromatin immunoprecipitation (ChIP) method. Nature Protocols-Electronic Edition.

[74] Nelson JD, Denisenko O, Sova P, Bomsztyk K (2006). Fast chromatin immunoprecipitation assay. Nucleic Acids Res.

[75] Robinson MD, McCarthy DJ, Smyth G (2010). K. edgeR: a Bioconductor package for differential expression analysis of digital gene expression data. Bioinformatics.

