# Supplementary material for: HIV-1 envelope glycoprotein stimulates viral transcription and increases the infectivity of the progeny virus through the manipulation of cellular machinery
Source: Sci Rep. 2017 Aug 25;7:9487. doi: 10.1038/s41598-017-10272-7 (PMC5573355; doi:10.1038/s41598-017-10272-7)
Supplement: Supplementary file 1 — Supplementary Information [file 41598_2017_10272_MOESM1_ESM.pdf]

HIV-1 envelope glycoprotein stimulates viral transcription and increases the infectivity of the progeny virus through the manipulation of cellular machinery

Xiaozhuo Ran , Zhujun Ao , Adriana Trajtman , Wayne Xu , Gary Kobinger , Yoav Keynan , and Xiaojian Yao

Supplementary figure: Uncropped full-length pictures of western blotting membranes

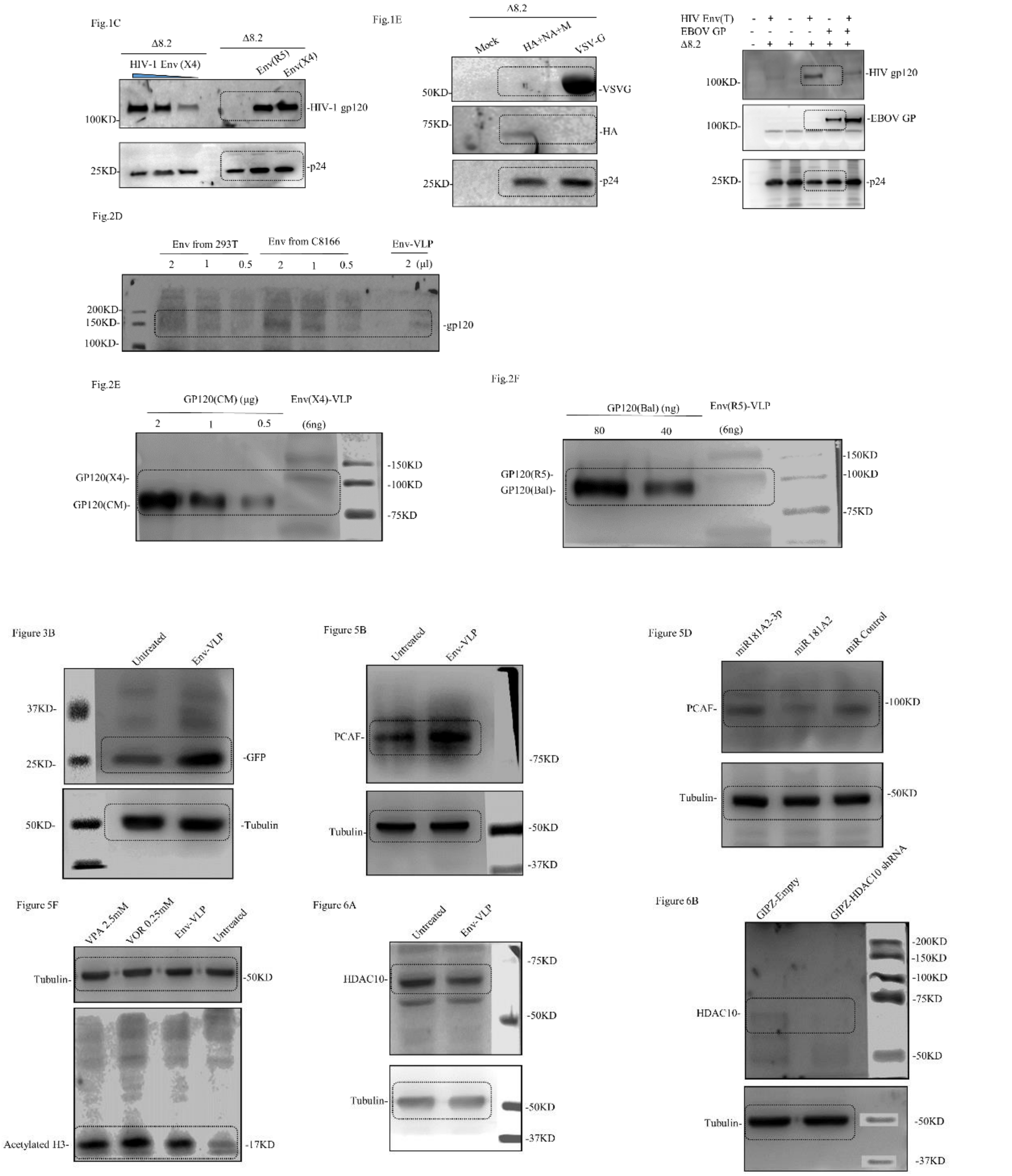

Uncropped full-length pictures of Western blotting membranes presented in the main figures. Membranes were often cut to enable blotting for multiple antibodies and to enable blotting for target proteins.
